# Development and external validation of a composite biomarker-based machine learning model for sarcopenia risk stratification in patients with cardiovascular disease

**DOI:** 10.3389/fcvm.2026.1814149

**Published:** 2026-07-09

**Authors:** Pengcheng Mei, Tao Ying, Jing Wu, Han Wang

**Affiliations:** 1 Department of Geriatrics, The Third People's Hospital of Chengdu (Affiliated Hospital of Southwest Jiaotong University), College of Medicine, Southwest Jiaotong University, Chengdu, Sichuan, China; 2 Department of Cardiology, The Third People's Hospital of Chengdu (Affiliated Hospital of Southwest Jiaotong University), College of Medicine, Southwest Jiaotong University, Chengdu, Sichuan, China

**Keywords:** cardiovascular diseases, composite biomarkers, machine learning, risk stratification, sarcopenia

## Abstract

**Background:**

Sarcopenia is common in patients with cardiovascular disease (CVD) and is associated with functional decline and adverse clinical outcomes. However, practical tools for early risk stratification in this population remain limited, particularly those incorporating composite metabolic biomarkers.

**Objective:**

To identify the most informative composite biomarker and the optimal machine learning (ML) algorithm for the development and validation of a sarcopenia risk stratification system in patients with CVD.

**Methods:**

We analyzed data from the China Health and Retirement Longitudinal Study (CHARLS, 2011–2015), the English Longitudinal Study of Ageing (ELSA, 2004–2013), and a hospital-based clinical cohort enrolled between 2023 and 2024. Cross-sectional analyses in CHARLS (*n* = 1,343) were used to examine the associations between candidate composite biomarkers and sarcopenia. Nine ML models were developed in a longitudinal CHARLS sub-cohort free of sarcopenia at baseline (*n* = 887), with hyperparameters optimized by 10-fold cross-validation. The best-performing CatBoost model incorporating TyG-BMI was further interpreted using Shapley Additive Explanations (SHAP) and externally validated in ELSA (*n* = 823) and the clinical cohort (*n* = 2,497), on the basis of which the Cardiovascular Disease–Sarcopenia Risk Score (CVD-SRS) was derived.

**Results:**

Among the evaluated composite biomarkers, TyG-BMI showed the highest discriminative ability for sarcopenia (AUC = 0.938). The CatBoost model showed good discrimination across cohorts, with AUCs of 0.982 in the training set, 0.907 in the internal validation set, 0.891 in ELSA, and 0.900 in the clinical cohort. The CVD-SRS provided consistent risk stratification across the internal and external validation cohorts.

**Conclusions:**

A CatBoost model integrating TyG-BMI showed good performance for sarcopenia risk stratification in patients with CVD. The CVD-SRS may facilitate early screening and help identify individuals who warrant further sarcopenia assessment in clinical practice.

## Introduction

Sarcopenia is a progressive skeletal muscle disorder characterized by the loss of muscle mass, strength, and physical performance, and is associated with falls, disability, hospitalization, and mortality (1). Its prevalence increases with age and is consistently higher in populations with chronic disease, making it an increasingly important public health concern. Cardiovascular disease (CVD), meanwhile, remains the leading cause of death worldwide and continues to impose a substantial burden in terms of morbidity, functional decline, and healthcare expenditure (2). In older adults, sarcopenia and CVD frequently coexist, but their coexistence is not merely incidental. The two conditions share several biological pathways, including chronic inflammation, insulin resistance, oxidative stress, and reduced anabolic reserve (3–5). More importantly, when sarcopenia develops in patients with CVD, it may further compromise functional capacity, aggravate frailty, and worsen prognosis (6). Previous studies have shown that sarcopenia affects up to one-third of patients with CVD (7), and in heart failure populations it has been associated with substantially increased risks of major adverse cardiovascular events and mortality (8). Early recognition of sarcopenia in CVD, therefore, is not simply a matter of identifying a coexisting geriatric syndrome. It is directly relevant to prognostic assessment, risk management, and the timely initiation of preventive interventions (9).

Current diagnosis of sarcopenia relies on established consensus frameworks, including those proposed by the Asian Working Group for Sarcopenia (AWGS) (10) and the European Working Group on Sarcopenia in Older People (EWGSOP) (11). These approaches emphasize the assessment of muscle strength, muscle mass, and physical performance, using measures such as handgrip strength, gait speed, dual-energy x-ray absorptiometry (DXA), and bioelectrical impedance analysis (BIA) (12, 13). Although these methods are clinically informative, they are not always well suited to routine cardiovascular practice or large-scale screening. Formal assessment often requires dedicated equipment, trained personnel, and standardized functional testing, which may be difficult to implement consistently in busy inpatient services, outpatient clinics, and resource-limited settings. As a result, sarcopenia may remain underrecognized until physical decline has become clinically evident. For patients with CVD, however, waiting for overt functional deterioration may mean missing the window for earlier intervention. A practical tool that enables timely risk stratification could help identify those who warrant formal sarcopenia evaluation and closer preventive management.

In this context, blood-based and composite metabolic biomarkers have emerged as promising candidates for early risk assessment (14). Studies show that elevated high-sensitivity C-reactive protein (hsCRP) levels are associated with lower appendicular lean mass (ALM) and decreased handgrip strength among older women (15). Furthermore, a randomized controlled trial conducted in a Taiwanese community hospital confirmed that several biomarkers, such as carnosine, creatinine, and uric acid, exhibit strong diagnostic performance for sarcopenia (16). Among emerging candidates, the triglyceride-glucose body mass index (TyG-BMI) is of particular interest because it integrates information on insulin resistance, adiposity, and metabolic reserve, each of which is closely related to the pathophysiology of sarcopenia in CVD (17). Yet the current evidence base remains limited. Most studies have been cross-sectional, have focused on single biomarkers, or have been conducted in relatively homogeneous populations, which constrains both generalizability and clinical translation.

Machine learning (ML) offers a potentially useful framework for addressing this problem. By accommodating nonlinear associations and interactions among clinical variables, ML models can integrate multidimensional biomarker information more flexibly than conventional approaches (18–20). Even so, ML-based sarcopenia risk stratification in patients with CVD has been insufficiently explored, particularly in the context of multicohort external validation across heterogeneous populations and healthcare settings. To address this gap, we developed and validated the Cardiovascular Disease−Sarcopenia Risk Score (CVD-SRS), an interpretable ML-based tool for sarcopenia risk stratification in patients with CVD. Using multicohort data, we aimed to identify the most informative composite biomarker, compare candidate ML algorithms, and develop a practical framework for early sarcopenia risk assessment in this high-risk population.

## Methods

### Study design and participants

**Figure 1:**
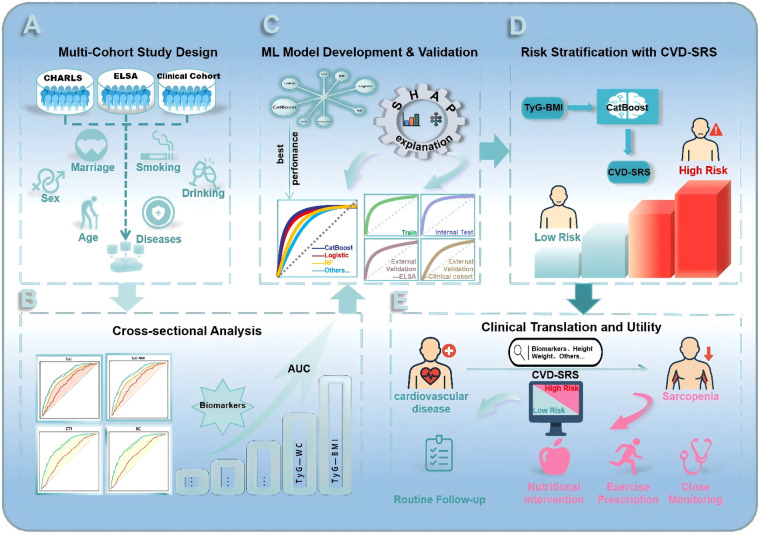
Study workflow and analytical framework. **(A)** Multicohort study design integrating demographic, lifestyle, and clinical data from CHARLS, ELSA, and the clinical cohort. **(B)** Cross-sectional evaluation of composite biomarkers for sarcopenia discrimination. **(C)** Machine learning model development and validation, with CatBoost selected as the final model and interpreted using SHAP. **(D)** Risk stratification based on the CatBoost-derived CVD-SRS. **(E)** Clinical application of the CVD-SRS for follow-up, intervention, and risk monitoring.

The overall analytical framework of the study is summarized in Figure 1. This multicohort study drew on three independent data sources: the China Health and Retirement Longitudinal Study (CHARLS) (21), the English Longitudinal Study of Ageing (ELSA) (22), and a hospital-based clinical cohort from Chengdu Third People's Hospital (Ethics ID: 2025-S-358). CHARLS was used for cross-sectional biomarker evaluation and longitudinal model development, ELSA for cross-national external validation, and the clinical cohort for real-world external validation. Although these cohorts differed in demographic composition, care setting, and sarcopenia assessment framework, they shared a common focus on patients with cardiovascular disease and provided comparable information on the key variables used in model development. Our aim was not to treat them as identical populations, but to examine whether the model retained useful performance across related but non-identical settings.

In CHARLS, participants with cardiovascular disease (CVD) and complete sarcopenia assessment data from the 2011 baseline survey were included in the cross-sectional analysis. For longitudinal model development, we further identified participants with CVD who were free of sarcopenia at baseline and had available follow-up data through 2015. This development cohort was randomly divided into a training set and an internal validation set at a ratio of 7:3.

**Figure 2:**
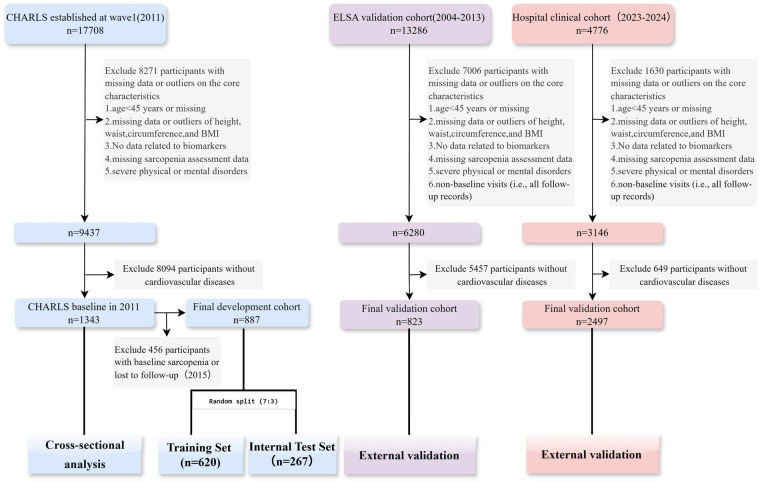
Flowchart of participant selection and cohort construction.

External validation was performed in two independent cohorts representing different population and care contexts. ELSA included community-dwelling adults aged 45 years or older with CVD, whereas the clinical cohort comprised hospitalized older adults with documented CVD enrolled between 2023 and 2024. In CHARLS and ELSA, CVD was defined on the basis of self-reported physician diagnosis of heart disease or cerebrovascular disease. In the clinical cohort, CVD was identified from discharge diagnoses coded according to ICD-10. The cohort selection process is shown in Figure 2, and detailed eligibility criteria for each cohort are provided in Supplementary Materials (Supplementary Appendix S1).

### Data collection and candidate predictors

Candidate predictors were selected on the basis of prior evidence, biological plausibility, and clinical relevance to sarcopenia risk in patients with cardiovascular disease (CVD). These included demographic characteristics (age and sex), marital status, lifestyle factors (smoking and alcohol consumption), comorbidities (hypertension and diabetes), and composite metabolic biomarkers.

Data collection followed standardized procedures within each cohort. In the population-based cohorts, participants underwent face-to-face interviews, physical examinations, and laboratory testing according to established survey protocols. In the clinical cohort, corresponding information was extracted from the electronic medical record and admission assessment forms. To improve comparability across datasets, variables were harmonized according to prespecified definitions. Only variables that could be defined in a reasonably consistent manner across cohorts were retained in the final modeling framework. Marital status was categorized as married or other, whereas smoking and drinking status were each classified as never or ever, with the latter including both former and current exposure.

Anthropometric measurements included height, weight, and waist circumference. Height and weight were measured with participants wearing light indoor clothing and no shoes, and waist circumference was measured at the level of the iliac crest at the end of normal expiration. Fasting blood samples were analyzed in central laboratories within the respective cohorts. The composite biomarkers evaluated in this study included the triglyceride-glucose index (TyG) (23, 24), TyG-body mass index (TyG-BMI), TyG-waist circumference (TyG-WC) (25), TyG-weight-adjusted waist index (TyG-WWI), TyG-waist-to-height ratio (TyG-WHtR) (26), the C-reactive protein–triglyceride-glucose index (CTI), cardiometabolic index (CMI), non-high-density lipoprotein cholesterol (non-HDL-C), remnant cholesterol (RC), and the non-high-density lipoprotein cholesterol to high-density lipoprotein cholesterol ratio (NHHR). Detailed variable definitions, harmonization procedures, and biomarker formulas are provided in Supplementary Materials (Supplementary Appendix S2).

### Outcome definition

Sarcopenia was defined according to the original diagnostic framework used in each cohort. The 2019 Asian Working Group for Sarcopenia (AWGS 2019) criteria were applied in CHARLS and the clinical cohort (27), whereas the revised European Working Group on Sarcopenia in Older People criteria (EWGSOP2) were used in ELSA (28). Across cohorts, the diagnosis incorporated the core domains of low muscle strength and low muscle mass, although the methods used to assess muscle mass, physical performance, and related cut-off values differed between datasets. We retained the original cohort-specific definitions rather than imposing a *post hoc* harmonized definition, because the available diagnostic components and measurement approaches were not identical across cohorts. This approach was considered more appropriate for preserving internal validity within each dataset. Detailed diagnostic components, muscle mass assessment methods, and cut-off values across the three cohorts are summarized in Supplementary Appendix Table S1.

### Cross-sectional association analysis

In the CHARLS cross-sectional cohort, multivariable logistic regression models were used to examine associations between individual composite biomarkers and prevalent sarcopenia. Three progressively adjusted models were fitted: an unadjusted model; a model adjusted for age, sex, and marital status; and a fully adjusted model further including smoking, alcohol consumption, hypertension, and diabetes. Sex-stratified analyses were performed separately. Results are presented as odds ratios (ORs) with 95% confidence intervals (CIs). Receiver operating characteristic (ROC) curves and the area under the curve (AUC) were used to assess the ability of individual composite biomarkers to distinguish participants with and without sarcopenia.

### Model development and validation

Prediction models were developed using the longitudinal CHARLS cohort of participants with CVD who were free of sarcopenia at baseline. Nine machine learning algorithms were evaluated: Logistic Regression, Random Forest, Decision Tree, Light Gradient Boosting Machine, CatBoost, Support Vector Machine, Multi-Layer Perceptron, Naive Bayes, and K-Nearest Neighbors Classifier. Candidate predictors included composite biomarkers together with demographic variables, lifestyle factors, and comorbidities. Hyperparameters were optimized by grid search with stratified 10-fold cross-validation in the training set.Final model selection was based on overall performance across the training, internal validation, and external validation datasets, with particular attention to discrimination, specificity, and performance stability.

Model performance was evaluated using ROC curves, AUCs, accuracy, sensitivity, specificity, positive predictive value (PPV), negative predictive value (NPV), and F1-score. Because the outcome prevalence was relatively low, precision-recall (PR) curves were also examined to better characterize discrimination under class imbalance. External validation was conducted in ELSA and in the clinical cohort to assess model generalizability across different populations and healthcare settings. Additional internal stability of the final model was evaluated using 10-fold cross-validation, and calibration plots were generated to assess agreement between predicted and observed risk in the training and internal validation sets.

### Model interpretation

To improve interpretability, Shapley Additive Explanations (SHAP) were used to quantify feature contributions to model predictions at both the population and individual levels (29). SHAP summary plots were used to rank predictors according to their overall importance, beeswarm plots were used to visualize the direction and magnitude of feature effects, and force plots were used to illustrate how specific variables, including TyG-BMI, contributed to individual-level predictions.

### Derivation of the CVD-SRS

Based on the best-performing model, we derived the Cardiovascular Disease–Sarcopenia Risk Score (CVD-SRS), defined as the model-predicted probability of sarcopenia ranging from 0 to 1. Higher values indicated greater estimated risk. A cut-off value was selected in the training set by maximizing the Youden index, allowing participants to be classified into low-risk and high-risk groups across validation cohorts. The CVD-SRS was developed as a practical tool for sarcopenia risk stratification in patients with CVD. Using routinely available clinical variables and composite biomarkers such as TyG-BMI, the model provides an individualized risk estimate that may support further sarcopenia assessment and preventive management.

### Statistical analysis

Continuous variables are presented as mean (standard deviation) or median (interquartile range), and categorical variables as counts (percentages). The Shapiro–Wilk test was used to assess normality. Group comparisons were performed using the independent-samples t test, Mann–Whitney *U*-test, chi-square test, or Fisher's exact test, as appropriate. Continuous variables were screened for outliers using Tukey's method (with a threshold of 1.5 × IQR) (30), and missing covariate data were handled using multiple imputation by chained equations (31). In the cross-sectional analysis, multivariable logistic regression models were used to evaluate associations between composite biomarkers and prevalent sarcopenia, with results expressed as odds ratios (ORs) and 95% confidence intervals (CIs). ROC curves and AUCs were generated to assess the discriminative performance of individual composite biomarkers. Pairwise comparisons of AUCs across candidate composite biomarkers in the training set were performed using DeLong's test. For model evaluation, performance metrics included AUC, accuracy, sensitivity, specificity, positive predictive value, negative predictive value, and F1-score. Because outcome prevalence was relatively low, precision-recall (PR) curves were also examined. Calibration plots were generated to assess agreement between predicted and observed risk. Decision curve analysis (DCA), net reclassification improvement (NRI), and integrated discrimination improvement (IDI) were used in additional analyses to assess potential clinical utility and incremental predictive value (32). All analyses were conducted using Python 3.12 and R 4.4.2, and all tests were two-sided with *P* < 0.05 considered statistically significant.

## Results

### Baseline characteristics of the study cohorts

The overall study workflow is shown in Figure 1, and participant selection is presented in Figure 2. The study included three independent cohorts: the CHARLS development cohort, the ELSA external validation cohort, and the clinical external validation cohort from Chengdu Third People's Hospital. In the CHARLS cross-sectional cohort, 1,343 participants with CVD were included (148 with sarcopenia and 1,195 without sarcopenia). ELSA included 823 participants (25 with sarcopenia and 798 without sarcopenia), and the clinical cohort included 2,497 participants (443 with sarcopenia and 2,054 without sarcopenia). For longitudinal model development, 887 CHARLS participants without baseline sarcopenia were identified and randomly divided into a training set (*n* = 620) and an internal validation set (*n* = 267).

To assess potential selection bias in the CHARLS source population, we compared included and excluded participants with CVD (Supplementary Table S1). Although excluded participants had much more missing biomarker data, the two groups were otherwise similar in their main demographic and clinical characteristics, with only small differences in most available variables. Overall, this suggests that the included CHARLS sample was broadly representative of the source CVD population, and that the larger differences in biomarker-related variables were mainly driven by data incompleteness among excluded participants. Baseline characteristics are summarized in Supplementary Tables S2–S5. Across all three cohorts, participants with sarcopenia were older and less likely to be married than those without sarcopenia. Most TyG-related and lipid-related composite biomarkers were lower in the sarcopenia group, with generally consistent patterns across cohorts. In addition, the training and internal validation sets showed broadly comparable baseline characteristics, supporting the suitability of the random split for model development.

### Associations between composite biomarkers and sarcopenia in the cross-sectional cohort

In the CHARLS cross-sectional cohort, most composite biomarkers were inversely associated with sarcopenia in both unadjusted and adjusted models (Table 1). In the overall analysis, these associations remained statistically significant after adjustment for age, sex, marital status, smoking, alcohol consumption, hypertension, and diabetes. In the fully adjusted model, the corresponding odds ratios were 0.56 (95% CI: 0.41–0.76) for TyG, 0.94 (95% CI: 0.93–0.95) for TyG-BMI, 0.98 (95% CI: 0.97–0.99) for TyG-WWI, 0.99 (95% CI: 0.99–0.99) for TyG-WC, and 0.37 (95% CI: 0.29–0.46) for TyG-WHtR (all *P* < 0.001). Similar inverse associations were also observed for CTI, CMI, non-HDL-C, NHHR, and RC.

**Table 1:** Multivariable associations between composite biomarkers and sarcopenia in the CHARLS cohort.

Variables	CHARLS								
	Model 1			Model 2			Model 3		
	OR (95%CI) *p*-value			OR (95%CI) *p*-value			OR (95%CI) *p*-value		
	Male	Female	Total	Male	Female	Total	Male	Female	Total
TyG	0.50 (0.33, 0.76) 0.001	0.50 (0.35, 0.72) <0.001	0.50 (0.38, 0.65) <0.001	0.59 (0.38, 0.93) 0.022	0.46 (0.31, 0.68) <0.001	0.52 (0.38, 0.69) <0.001	0.68 (0.42, 1.11) 0.123	0.48 (0.31, 0.72) <0.001	0.56 (0.41, 0.76) <0.001
TyG-BMI	0.93 (0.91, 0.95) <0.001	0.95 (0.94, 0.96) <0.001	0.94 (0.94, 0.95) <0.001	0.93 (0.91, 0.95) <0.001	0.95 (0.94, 0.96) <0.001	0.94 (0.93, 0.95) <0.001	0.92 (0.90, 0.94) <0.001	0.95 (0.93, 0.96) <0.001	0.94 (0.93, 0.95) <0.001
TyG-WWI	0.98 (0.97, 1.00) 0.027	0.99 (0.98, 1.00) 0.160	0.99 (0.98, 1.00) 0.008	0.98 (0.96, 1.00) 0.021	0.98 (0.97, 0.99) 0.002	0.98 (0.97, 0.99) <0.001	0.98 (0.96, 1.00) 0.030	0.98 (0.97, 0.99) 0.005	0.98 (0.97, 0.99) <0.001
TyG-WC	0.99 (0.99, 1.00) <0.001	0.99 (0.99, 1.00) <0.001	0.99 (0.99, 0.99) <0.001	0.99 (0.99, 0.99) <0.001	0.99 (0.99, 0.99) <0.001	0.99 (0.99, 0.99) <0.001	0.99 (0.99, 0.99) <0.001	0.99 (0.99, 0.99) <0.001	0.99 (0.99, 0.99) <0.001
TyG-WHtR	0.36 (0.26, 0.51) <0.001	0.48 (0.38, 0.61) <0.001	0.44 (0.37, 0.53) <0.001	0.32 (0.22, 0.47) <0.001	0.38 (0.29, 0.49) <0.001	0.36 (0.29, 0.45) <0.001	0.32 (0.22, 0.46) <0.001	0.37 (0.28, 0.48) <0.001	0.37 (0.29, 0.46) <0.001
CTI	0.67 (0.49, 0.92) 0.013	0.63 (0.48, 0.83) 0.001	0.65 (0.52, 0.79) <0.001	0.69 (0.49, 0.98) 0.036	0.55 (0.41, 0.75) <0.001	0.61 (0.48, 0.76) <0.001	0.79 (0.55, 1.15) 0.221	0.57 (0.41, 0.78) <0.001	0.65 (0.51, 0.83) <0.001
CMI	0.49 (0.34, 0.70) <0.001	0.57 (0.43, 0.74) <0.001	0.53 (0.43, 0.65) <0.001	0.55 (0.38, 0.78) <0.001	0.52 (0.39, 0.68) <0.001	0.53 (0.42, 0.65) <0.001	0.58 (0.40, 0.82) 0.003	0.52 (0.39, 0.69) <0.001	0.55 (0.44, 0.68) <0.001
Non-hdlc	0.99 (0.98, 1.00) 0.005	0.99 (0.99, 1.00) 0.069	0.99 (0.99, 1.00) <0.001	0.99 (0.99, 1.00) 0.095	0.99 (0.99, 1.00) 0.015	0.99 (0.99, 1.00) 0.003	1.00 (0.99, 1.01) 0.675	0.99 (0.99, 1.00) 0.036	0.99 (0.99, 1.00) 0.014
NHHR	0.64 (0.51, 0.80) <0.001	0.66 (0.54, 0.82) <0.001	0.65 (0.56, 0.76) <0.001	0.69 (0.55, 0.87) 0.002	0.60 (0.48, 0.75) <0.001	0.64 (0.55, 0.76) <0.001	0.72 (0.57, 0.92) 0.009	0.61 (0.48, 0.76) <0.001	0.67 (0.57, 0.79) <0.001
RC	0.96 (0.94, 0.98) <0.001	0.99 (0.97, 1.00) 0.023	0.98 (0.97, 0.99) <0.001	0.97 (0.95, 0.99) 0.004	0.98 (0.97, 1.00) 0.011	0.98 (0.97, 0.99) <0.001	0.97 (0.95, 0.99) 0.013	0.98 (0.97, 1.00) 0.027	0.98 (0.97, 0.99) 0.001

OR, odds ratio; CI, confidence interval.

Model 1: was unadjusted.

Model 2: was adjusted for age, sex, and marital status in the overall analysis.

Model 3: was further adjusted for smoking, alcohol consumption, hypertension, and diabetes.

In sex-stratified analyses, sex was not included as a covariate.

Sex-stratified analyses showed that the inverse associations of TyG-BMI, TyG-WC, and TyG-WHtR with sarcopenia were generally consistent in both men and women, although the magnitude of association varied across biomarkers. Overall, the direction of association remained stable across models and sex strata, suggesting that the observed relationships were not fully explained by demographic characteristics, lifestyle factors, or major comorbidities.

Supplementary Figure S1 and Supplementary Table S6 show the discriminative performance of the evaluated composite biomarkers for sarcopenia across the three adjustment models. Among them, TyG-BMI had the highest AUC in the fully adjusted model (0.938, 95% CI: 0.922–0.954), followed by TyG-WC (0.878, 95% CI: 0.853–0.903).

### Comparison and validation of machine learning models

**Figure 3:**
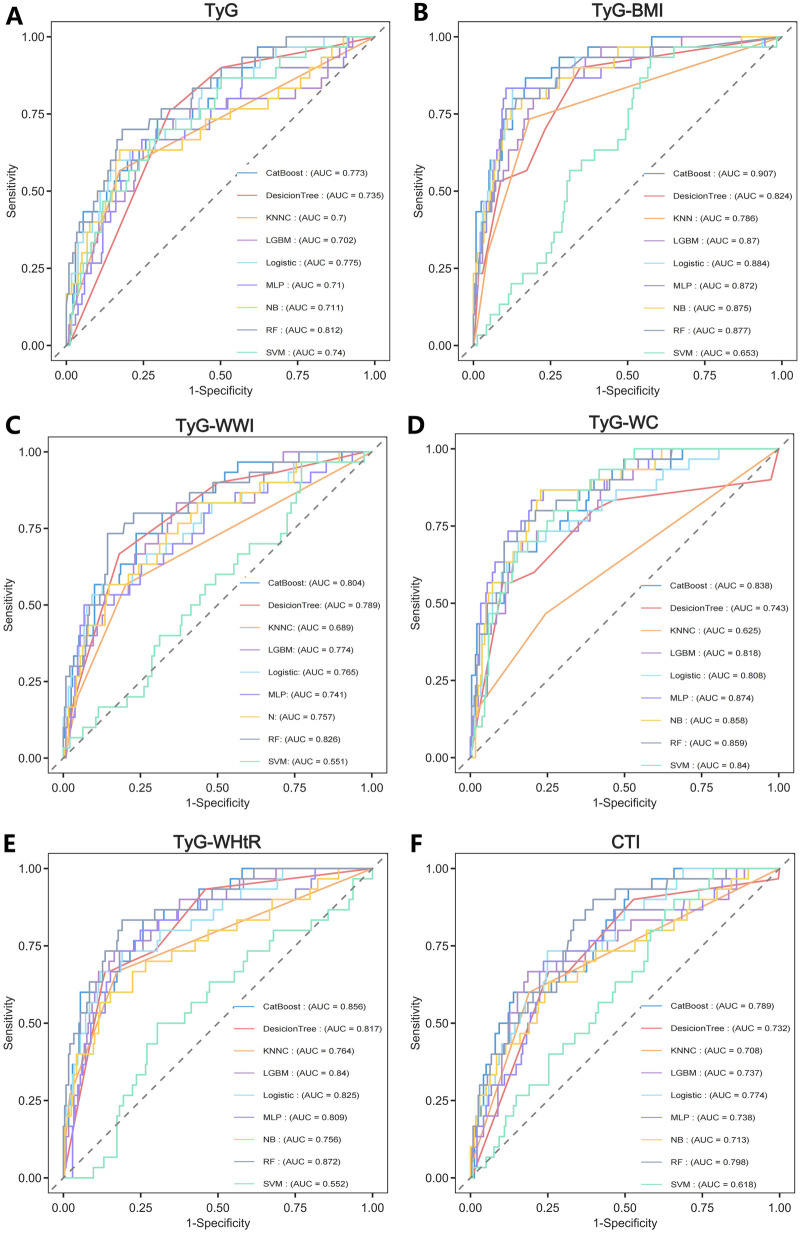
Comparison of ROC curves across the nine machine learning models. Panels (A–J) represent TyG, TyG-BMI, TyG-WWI, TyG-WC, TyG-WHtR, CTI, CMI, Non-HDL-C, NHHR, and RC, respectively. Each curve corresponds to one candidate model (CatBoost, DecisionTree, KNNC, LGBM, Logistic, MLP, NB, RF, and SVM). AUC values are shown in the legends.

Nine machine learning algorithms were developed and evaluated in the longitudinal CHARLS cohort. Their ROC curves in the development dataset are shown in Figure 3. In the training set, CatBoost showed the highest AUC among the clinically interpretable candidate models, whereas LGBM achieved near-perfect training performance, suggesting possible overfitting. In the internal validation set, CatBoost yielded the highest AUC (0.907) and maintained high specificity (0.996). In the external validation cohorts, some alternative models showed comparable or slightly higher AUCs in individual datasets; however, CatBoost retained consistently good discrimination, high specificity, and stable performance across the full development and validation framework. On this basis, CatBoost was selected as the final model. Detailed performance metrics for all candidate models are presented in Supplementary Tables S7, S8.

**Figure:**
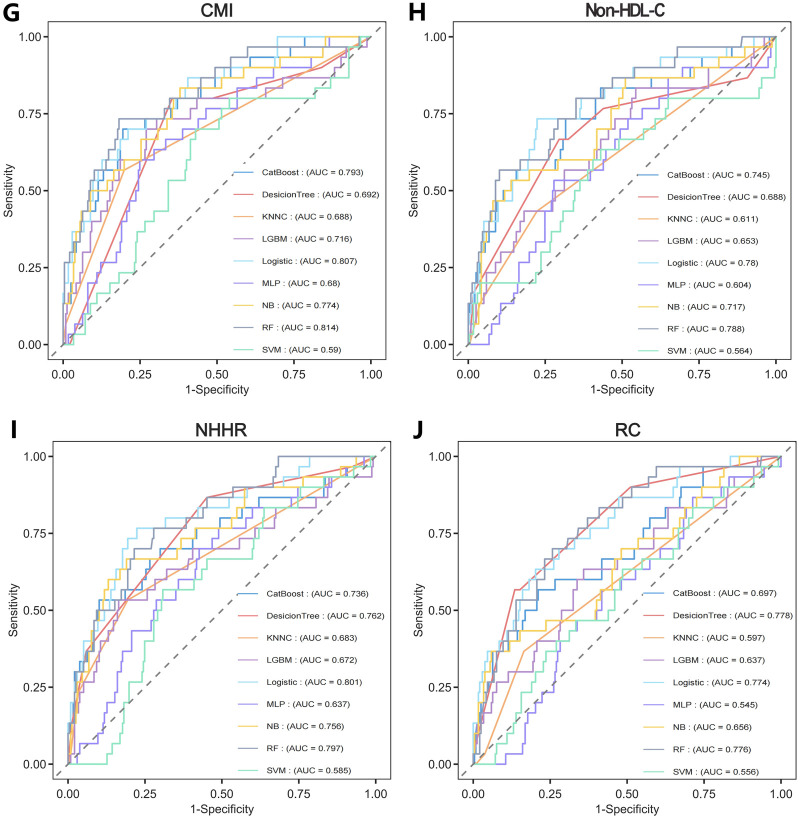


**Figure 4:**
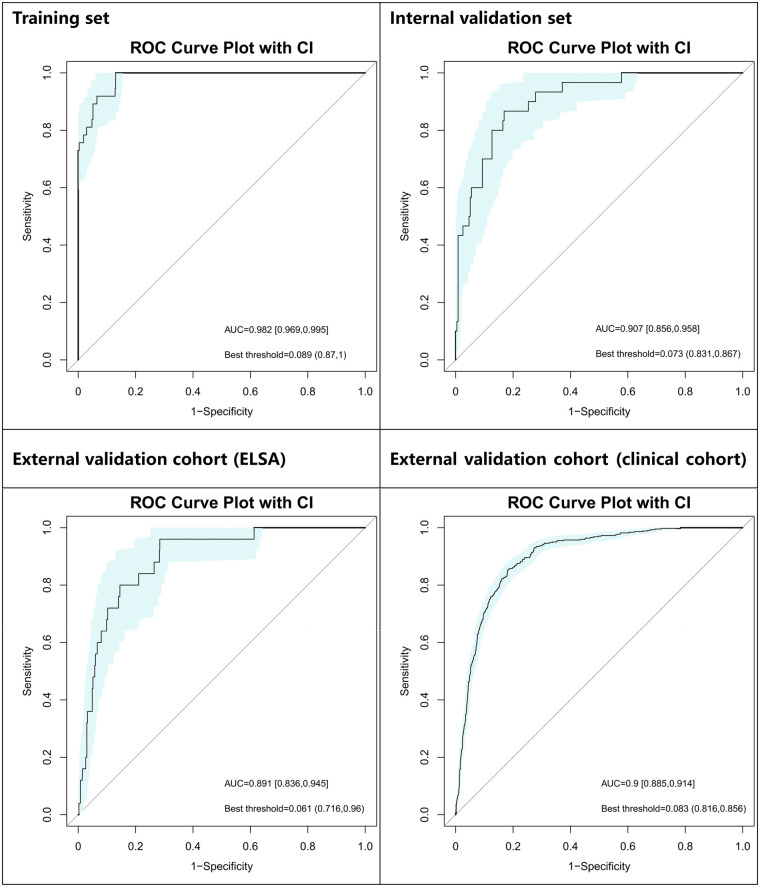
Discriminative performance of the final CatBoost model across the development and validation cohorts. ROC curves are shown for the final CatBoost model in the training set, internal validation set, ELSA external validation cohort, and clinical external validation cohort. Shaded areas indicate 95% confidence intervals. The threshold shown in each panel corresponds to the cutoff used for classification, with the values in parentheses indicating sensitivity and specificity.

In the training set, the CatBoost model achieved an AUC of 0.982 (95% CI: 0.969–0.995), with an accuracy of 0.966 and specificity of 1.000. In the internal validation set, the AUC was 0.907 (95% CI: 0.856–0.959), with an accuracy of 0.895 and specificity of 0.996. External validation showed similarly good discrimination, with AUCs of 0.891 (95% CI: 0.836–0.945) in ELSA and 0.900 (95% CI: 0.885–0.914) in the clinical cohort (Figure 4).

Model performance varied across validation cohorts according to outcome prevalence. In ELSA, where sarcopenia prevalence was low (3.0%), the model retained high accuracy (0.965), specificity (0.994), and negative predictive value (0.971), whereas the positive predictive value and F1-score were lower (0.167 and 0.065, respectively). In the clinical cohort, where sarcopenia prevalence was higher (17.7%), the positive predictive value increased to 0.634, while specificity remained high at 0.987. Additional internal assessment using 10-fold cross-validation yielded a mean AUC of 0.877, mean accuracy of 0.934, mean specificity of 0.993, and mean negative predictive value of 0.940 (Supplementary Table S9), supporting the overall stability of the final model.

Additional model evaluation using precision-recall curves, decision curve analysis, and calibration plots is presented in Supplementary Figure S2. These analyses suggested stronger overall performance in the training set and more moderate performance in the internal validation set, particularly with respect to calibration and precision-recall characteristics. Taken together, these findings indicate that the final CatBoost model achieved strong discrimination, while its calibration-related performance was more conservative in the validation setting.

### Incremental value of TyG-BMI and model interpretation

Pairwise comparisons using DeLong's test showed that TyG-BMI had a higher AUC than each of the other evaluated composite biomarkers (Supplementary Table S10). The smallest AUC difference was observed between TyG-BMI and TyG-WHtR (difference = 0.051, 95% CI: 0.036–0.066), whereas the largest difference was observed between TyG-BMI and RC (difference = 0.211, 95% CI: 0.154–0.268). Additional analyses showed that both NRI and IDI were consistently greater than zero when the final CatBoost model incorporating TyG-BMI was compared with models based on other composite biomarkers (Supplementary Table S11), supporting the incremental predictive value of TyG-BMI.

SHAP analysis further highlighted the importance of TyG-BMI in the final model. TyG-BMI had the largest mean absolute SHAP value (0.868), followed by age (0.585) (Supplementary Figure S3). Higher TyG-BMI values were associated with lower predicted sarcopenia risk, whereas older age was associated with higher predicted risk.

### Performance of the CVD-SRS

The Cardiovascular Disease–Sarcopenia Risk Score (CVD-SRS) was significantly associated with sarcopenia across the development and validation cohorts (Table 2). In the CHARLS training set, the CVD-SRS was strongly associated with sarcopenia in both the unadjusted model (OR = 10.35, 95% CI: 5.99–20.82, *P* < 0.001) and the fully adjusted model (OR = 10.94, 95% CI: 6.11–23.67, *P* < 0.001). The association remained significant in the internal validation set, with adjusted ORs ranging from 3.36 to 3.87. Similar associations were observed in the external validation cohorts, with adjusted ORs of 2.12 in ELSA and 3.39 in the clinical cohort, indicating that the CVD-SRS retained its association with sarcopenia across different datasets.

**Table 2:** Associations of the CVD-SRS with sarcopenia across the development and validation cohorts.

Outcome		CHARLS			ELSA	Clinical Cohort
		Total	Training set	Internal validation set	External validation set	External validation set
Model 1	OR (95% CI) *P*	6.057 (4.420,8.673) <0.001	10.347 (5.989,20.816) <0.001	3.865 (2.616,6.076) <0.001	1.981 (1.610,2.446) <0.001	3.822 (3.374,4.355) <0.001
Model 2	OR (95% CI) *P*	5.804 (4.203,8.401) <0.001	10.507 (5.956,22.214) <0.001	3.405 (2.225,5.540) <0.001	1.951 (1.551,2.467) <0.001	3.336 (2.930,3.820) <0.001
Model 3	OR (95% CI) *P*	5.819 (4.176,8.511) <0.001	10.938 (6.108,23.674) <0.001	3.359 (2.116,5.678) <0.001	2.124 (1.658,2.758) <0.001	3.392 (2.969,3.899) <0.001

CVD-SRS, cardiovascular disease–sarcopenia risk score; OR, odds ratio; CI, confidence interval; CHARLS, China health and retirement longitudinal study; ELSA, English longitudinal study of ageing.

Model 1: was unadjusted.

Model 2: was adjusted for age, sex, and marital status.

Model 3: was further adjusted for hypertension and diabetes.

Restricted cubic spline analysis showed a nonlinear association between the continuous CVD-SRS and sarcopenia risk, with risk increasing more steeply at higher score levels (Supplementary Figure S5). Additional analyses also showed that higher age was associated with greater predicted risk, whereas being married was associated with lower predicted risk (Supplementary Table S12 and Supplementary Figure S4). Based on the maximum Youden index in the training set, participants were classified as low risk (<0.0892) or high risk (≥0.0892).

## Discussion

To our knowledge, this is the first multicohort study to develop and externally validate an interpretable machine learning framework for sarcopenia risk stratification in patients with cardiovascular disease (CVD). Several findings deserve emphasis. First, the TyG index and its derived composite biomarkers were inversely associated with sarcopenia in patients with CVD, with TyG-BMI showing the strongest and most consistent association. Second, among the biomarkers examined, TyG-BMI showed the highest discriminative ability for sarcopenia. Third, incorporation of TyG-BMI into a CatBoost-based model yielded good performance across internal and external validation datasets. Finally, the resulting Cardiovascular Disease–Sarcopenia Risk Score (CVD-SRS) remained significantly associated with sarcopenia across cohorts, supporting its potential use for risk stratification in this high-risk population.

The clinical importance of this work lies in the fact that sarcopenia in patients with CVD is not simply a coexisting age-related condition, but one with meaningful prognostic implications. Cardiovascular disease remains a leading cause of mortality worldwide, while sarcopenia contributes substantially to disability, functional decline, and health burden (33). Their coexistence has been associated with poorer outcomes, including reduced functional capacity and increased mortality (34, 35). A Japanese cross-sectional study of hospitalized patients with type 2 diabetes reported that low muscle mass was independently associated with a higher risk of CVD in individuals with visceral adiposity (36). These observations reinforce the need for timely identification of sarcopenia in patients with cardiovascular disease (37). In this context, our findings support the view that early risk stratification may help identify patients who warrant more detailed sarcopenia assessment and earlier preventive management.

Recent years have seen increasing interest in the use of composite biomarkers for risk prediction (38–40). A study in older Chinese adults found that the C-reactive protein–triglyceride-glucose index (CTI) was associated with cardiovascular disease risk (41). Other work in metabolic syndrome populations has shown that TyG-related indices are associated with cause-specific mortality, with TyG-WC and TyG-WHtR showing particular relevance to cardiovascular death (42). In patients with type 2 diabetes mellitus, the TyG index has also been linked to a lower likelihood of low muscle mass (43). Furthermore, TyG-BMI and TyG-WC, which integrate metabolic information with anthropometric characteristics, have been reported to outperform the TyG index alone in predicting sarcopenia (44). Against this background, our study extends the literature by showing that several composite metabolic biomarkers, including TyG-BMI and NHHR, are inversely associated with sarcopenia in patients with CVD.

The inverse association between TyG-related indices and sarcopenia observed in our study is also consistent with several previous reports. Using adults aged 60 years and older from CHARLS, Chen et al. showed that the TyG index was significantly and inversely associated with sarcopenia in older Chinese adults (45). A Korean longitudinal study similarly suggested that higher metabolic indices may be protective against age-related muscle loss (46). In addition, a Chinese cross-sectional study of postmenopausal women found that higher TyG-BMI was associated with a lower likelihood of sarcopenia (47). Taken together, these findings suggest that TyG-BMI may capture clinically relevant variation in metabolic reserve and body composition more effectively than single biomarkers alone.

These observations should not be interpreted to mean that higher adiposity is uniformly protective. Rather, they invite a more careful reconsideration of the so-called obesity paradox in chronic disease (48). In some older adults, apparently normal body weight may conceal substantial muscle loss, whereas higher body mass may partly reflect preserved nutritional reserve or less advanced catabolic depletion. In a large observational study of chronic heart failure, obesity was associated with lower mortality, but malnutrition markedly increased the risk of death, including among patients with obesity (49). A systematic review further suggested that, among community-dwelling older adults, sarcopenic obesity may show a different survival pattern than sarcopenia alone (50). A likely explanation is that the balance between muscle and fat mass, rather than body weight itself, is central to metabolic health (51). From this perspective, our findings are more consistent with the importance of body composition and metabolic reserve than with a simplistic interpretation of obesity as beneficial.

This interpretation has practical implications. In patients with CVD, identifying those at higher risk of sarcopenia may support a more individualized approach to care. Management should not focus solely on weight reduction, but should also consider preservation of muscle mass and function. Nutritional strategies should aim to improve metabolic health while avoiding excessive caloric restriction that may aggravate muscle loss. Adequate protein intake remains important, and exercise prescriptions should ideally combine aerobic and resistance components to improve both cardiovascular fitness and muscle function (52). In this setting, repeated attention to body composition may be more informative than body weight alone, and unintentional weight loss should be regarded cautiously rather than automatically encouraged (53).

From a methodological perspective, machine learning offers advantages for integrating heterogeneous clinical and biomarker data, especially when relationships between predictors and outcomes are likely to be nonlinear (54). In the present analysis, CatBoost provided the most favorable overall balance of discrimination and stability across the development and validation datasets, even though some alternative models showed comparable performance in individual external cohorts. This pattern suggests that the choice of CatBoost was supported less by isolated superiority in a single dataset than by its more consistent behavior across the full modeling framework. By incorporating SHAP, we further improved interpretability and were able to show that TyG-BMI and age were the most influential predictors in the final model. This improves the transparency of the model and may facilitate clinical interpretation of individual predictions (55). Similar advantages of interpretable machine learning approaches have also been reported in other medical prediction settings (56). At the same time, the three cohorts used for external validation were not identical in population structure, care setting, or sarcopenia ascertainment. The validation results should therefore be understood as evidence of model performance across related but non-uniform settings, rather than as reproducibility under fully standardized conditions.

Several limitations should be acknowledged. First, although external validation was performed in both ELSA and a clinical cohort, model development relied primarily on Chinese data, which may limit generalizability to other ethnic and healthcare settings. Second, sarcopenia was defined according to the original framework used in each cohort rather than a single harmonized standard. CHARLS and the clinical cohort used AWGS 2019-based criteria, whereas ELSA used EWGSOP2-based criteria, and methods of muscle mass assessment also differed across datasets. These differences may have introduced some heterogeneity in case classification and therefore limit strict comparability of absolute prevalence and effect estimates across cohorts. They may also have contributed to some degree of cohort-related measurement bias in external validation. We retained the original cohort-specific definitions to preserve internal validity within each dataset, given the differences in available diagnostic components and measurement approaches. Third, because the analytic samples were derived after eligibility restriction and exclusion of participants with incomplete key data, some degree of selection bias cannot be excluded. Finally, the clinical validation cohort was derived from a single center, and further multicenter prospective validation is needed. Despite these limitations, the study also has important strengths. It integrated cross-sectional biomarker evaluation, longitudinal model development, and external validation in both community-based and hospital-based populations. The CVD-SRS is based on routinely available variables, which may enhance its practicality in settings where formal sarcopenia assessment is not always feasible. Future prospective studies are needed to further validate and refine the CVD-SRS and to determine whether its implementation can improve early identification and preventive management of sarcopenia in patients with CVD.

## Conclusions

Based on the best-performing CatBoost model, we developed the Cardiovascular Disease–Sarcopenia Risk Score (CVD-SRS) for risk stratification in patients with cardiovascular disease. The model showed good discrimination across the development and validation cohorts. As a tool based on routinely available variables, the CVD-SRS may support early identification of patients who warrant further sarcopenia assessment and preventive management. In clinical practice, it may help inform decisions about follow-up intensity, referral for formal evaluation, and allocation of supportive care resources. In settings where comprehensive sarcopenia assessment is not routinely available, this framework may also contribute to a more targeted use of available clinical resources. More broadly, it may support a more integrated approach to cardiovascular care by encouraging earlier attention to muscle health, nutritional status, and functional decline, with the aim of strengthening preventive management and improving overall patient outcomes.

## Data Availability

The raw data supporting the conclusions of this article will be made available by the authors, without undue reservation.
