# Supplementary material for: Development and external validation of a composite biomarker-based machine learning model for sarcopenia risk stratification in patients with cardiovascular disease
Source: Front Cardiovasc Med. 2026 Jul 9;13:1814149. doi: 10.3389/fcvm.2026.1814149 (PMC13391546; doi:10.3389/fcvm.2026.1814149)
Supplement: Supplementary file 1 [file Supplementaryfile1.docx]

**Supplementary appendix**

**Appendix 1. Detailed Participant Selection and Cohort Construction**

**The CHRALS cohort**

The China Health and Retirement Longitudinal Study (CHARLS) is a nationally representative longitudinal survey of middle-aged and older adults in China. The baseline survey (2011–2012) used a multistage, stratified, probability-proportional-to-size sampling design and enrolled 17,708 participants aged 45 years or older from 28 provinces, 150 counties or districts, and 450 villages or urban communities. Data collection was conducted by trained interviewers using standardized protocols, including face-to-face questionnaires, physical examinations, and biomarker assessments.

For the present study, CHARLS served as the source for both cross-sectional biomarker evaluation and longitudinal model development. Participant selection began with the 2011 baseline sample (n = 17,708). Individuals were excluded if they were younger than 45 years or had missing age information; had missing or implausible anthropometric data (including height, waist circumference, or body mass index); lacked key biomarker measurements; had incomplete sarcopenia assessment data; or were unable to complete the relevant assessments because of severe physical or mental conditions. After these exclusions, 9,437 participants remained.

Among these, 8,094 individuals without cardiovascular disease (CVD) were excluded, leaving 1,343 participants with CVD for the cross-sectional analysis. To construct the longitudinal development cohort, we further excluded 456 participants who had sarcopenia at baseline or were lost to follow-up before 2015. The final longitudinal cohort included 887 participants with CVD who were free of sarcopenia at baseline. These participants were randomly divided into a training set (n = 620) and an internal validation set (n = 267).

**The ELSA cohort**

The English Longitudinal Study of Ageing (ELSA) is an ongoing population-based panel study of older adults in England. Established in 2002–2003, ELSA was designed to examine the health, social, and economic circumstances of the English population and includes repeated biennial assessments. Data collection combines computer-assisted personal interviews, self-completion questionnaires, nurse visits, physical performance testing, anthropometric measurements, and blood sampling.

In this study, ELSA served as the cross-national external validation cohort. Data from Waves 2–6 (2004–2013) were considered because biomarker data were available from Wave 2 onward. The initial analytical sample comprised 13,286 observations. To improve comparability with the CHARLS baseline design, only the first eligible baseline observation for each participant was retained, and all non-baseline repeated records were excluded. Additional exclusions were applied for the following reasons: age below 45 years or missing age information; missing or implausible anthropometric measurements; unavailable core biomarker data; incomplete sarcopenia assessment; or severe physical or mental conditions preventing valid assessment. After these exclusions, 6,280 participants remained.

Among these, 5,457 individuals without CVD were excluded, yielding a final external validation cohort of 823 participants with CVD.

**The clinical cohort**

The clinical external validation cohort was derived from consecutive inpatient records from the Department of Geriatrics, Chengdu Third People’s Hospital, collected between 2023 and 2024. This real-world cohort was used to evaluate the performance of the final model in a hospital-based setting. The initial dataset comprised 4,776 hospitalized patients.

Participants were excluded if they were younger than 45 years or had incomplete demographic data; had missing or implausible anthropometric measurements; lacked laboratory data required for biomarker calculation; had insufficient data for sarcopenia assessment; had severe physical or mental disorders; or represented repeated hospitalizations during the study period, in which case only the first admission was retained. After these exclusions, 3,146 eligible participants remained.

CVD cases were then identified using discharge diagnoses coded according to the International Classification of Diseases, Tenth Revision (ICD-10). A total of 649 participants without CVD were excluded, resulting in a final clinical external validation cohort of 2,497 hospitalized older adults with CVD. All clinical data were de-identified before analysis, and the study was approved by the Institutional Review Board of Chengdu Third People’s Hospital (Ethics ID: 2025-S-358).

**Appendix 2: Definitions and Formulas of Composite Biomarkers**

**BMI^1^(Body Mass Index)** = Weight (kg) / Height (m)^2

**WC(Waist Circumference)** = measured in centimeters

**WWI(Weight-adjusted Waist Index)**=Waist Circumference (cm) / √Weight (kg)

**WHtR(Waist-to-Height Ratio)** = Waist Circumference (cm) / Height (cm)

**TyG ^2^(Triglyceride-Glucose Index)** = ln[Fasting Triglycerides (mg/dL) × Fasting Glucose (mg/dL)/ 2]

**TyG-BMI^3^**=TyG × BMI

**TyG-WWI^4^**=TyG × WWI

**TyG-WC^4^**=TyG × WC

**TyG- WHtR^4^**=TyG × WHtR

**CTI^5^(C-reactive protein Triglyceride Glucose Index)**=0.412 × ln(C-reactive protein (mg/L)) + ln(Fasting Triglycerides (mg/dL) × Fasting Glucose (mg/dL)) / 2

**CMI^6^(Cardiometabolic Index)**= [Fasting Triglycerides (mg/dL) / High density lipoprotein cholesterol (mg/dL)] × [Waist Circumference (cm) / Height (cm)]

**Non-hdlc^7^(Non-high density lipoprotein cholesterol)**=Total Cholesterol (mg/dL) - High density lipoprotein cholesterol (mg/dL)

**NHHR^8^(Non-high density lipoprotein cholesterol to High-density lipoprotein cholesterol Ratio)**=Non-hdlc(mg/dL)/hdlc(mg/dL)

**RC^6^(Remnant Cholesterol)**=Total Cholesterol (mg/dL) - High density lipoprotein cholesterol (mg/dL) - Low density lipoprotein cholesterol (mg/dL)

**eReferences：**

1. WHO Expert Consultation. Appropriate body-mass index for Asian populations and its implications for policy and intervention strategies. Lancet. 2004;363(9403):157-163.
2. Guerrero-Romero F, Simental-Mendía LE, González-Ortiz M, Martínez-Abundis E, Ramos-Zavala MG, Hernández-González SO, et al. The product of triglycerides and glucose, a simple measure of insulin sensitivity. Comparison with the euglycemic-hyperinsulinemic clamp. J Clin Endocrinol Metab. 2010;95(7):3347-3351.
3. Lee JW, Lim NK, Park HY. The product of fasting plasma glucose and triglycerides improves risk prediction of type 2 diabetes in middle-aged Koreans. BMC Endocr Disord. 2018;18(1):33.
4. Lim J, Kim J, Koo SH, Kwon GC. Comparison of triglyceride glucose index, and related parameters to predict insulin resistance in Korean adults: an analysis of the 2007-2010 Korean National Health and Nutrition Examination Survey. PLoS One. 2019;14(3):e0212963.
5. Zhang L, Li S, Liu D, Gui J, Hu J, Wang Q, Mao W. The relationship between C-reactive protein-triglyceride-glucose index and cardiovascular disease: insights from the China Health and Retirement Longitudinal Study (CHARLS). Cardiovasc Diabetol. 2025;24(1):410.
6. Wakabayashi I, Daimon T. The "cardiometabolic index" as a new marker determined by adiposity and blood lipids for discrimination of diabetes mellitus. Clin Chim Acta. 2015;438:274-278.
7. Quispe R, Martin SS, Michos ED, Lamba I, Blumenthal RS, Saeed A, et al. Remnant cholesterol predicts cardiovascular disease beyond LDL and ApoB: a primary prevention study. Eur Heart J. 2021;42(42):4324-4332.
8. Chen X, Liang M, Zhang J, Xu C, Chen L, Hu R, Zhong J. The non-high-density lipoprotein cholesterol to HDL-C ratio and its association with chronic kidney disease in Chinese adults with type 2 diabetes: a preliminary study. Nutrients. 2025;17(7):1125.

**Supplementary Table S1：Baseline comparison of included and excluded participants with cardiovascular disease in CHARLS**

| **Variable** | **Category** | **Included(N = 1343)** | **Excluded(N = 1159)** | **P value** | **SMD** |
| --- | --- | --- | --- | --- | --- |
| Age |  | 62 (56, 68) | 62 (55, 71) | 0.444 | 0.04 |
| Missing, n |  |  | 25 |  |  |
| Sex, n (%) | Male | 565 (42.1%) | 492 (42.5) | 0.892 | 0.01 |
|  | Female | 778 (57.9%) | 667 (57.5) |  |  |
| Missing, n |  | 0 | 0 |  |  |
| Marital status, n (%) | Yes | 1138 (84.7%) | 964 (83.2) | 0.226 | 0.05 |
|  | No | 205 (15.3%) | 195 (16.8) |  |  |
| Missing, n |  | 0 | 0 |  |  |
| TyG |  | 8.75 (8.34, 9.22) | 8.82 (8.37, 9.36) | 0.074 | 0.11 |
| Missing, n |  | 0 | 816 |  |  |
| TyG-BMI |  | 212 (183, 243) | 217 (185, 246) | 0.487 | 0.15 |
| Missing, n |  | 0 | 1,119 |  |  |
| TyG-WWI |  | 99 (91, 108) | 99 (90, 106) | 0.719 | -0.12 |
| Missing, n |  | 0 | 1,115 |  |  |
| TyG-WC |  | 766 (677, 860) | 757 (677, 862) | 0.767 | -0.10 |
| Missing, n |  | 0 | 1,112 |  |  |
| TyG-WHtR |  | 4.90 (4.29, 5.45) | 4.89 (4.42, 5.37) | 0.926 | -0.08 |
| Missing, n |  | 0 | 1,118 |  |  |
| CTI |  | 8.87 (8.32, 9.49) | 9.05 (8.45, 9.71) | 0.007 | 0.10 |
| Missing, n |  | 0 | 814 |  |  |
| CMI |  | 1.39 (0.78, 2.42) | 1.66 (0.95, 2.85) | 0.147 | 0.07 |
| Missing, n |  | 0 | 1,116 |  |  |
| Non-hdlc |  | 145 (118, 170) | 142 (117, 174) | 0.709 | -0.04 |
| Missing, n |  | 0 | 813 |  |  |
| NHHR |  | 3.07 (2.24, 4.21) | 3.14 (2.24, 4.23) | 0.732 | 0.01 |
| Missing, n |  | 0 | 814 |  |  |
| RC |  | 22 (13, 35) | 23 (12, 38) | 0.870 | 0.03 |
| Missing, n |  | 0 | 814 |  |  |
| Hypertension, n (%) | Yes | 867 (64.7) | 751 (64.9) | 0.821 | 0.01 |
|  | No | 473 (35.3) | 406 (35.1) |  |  |
| Missing, n |  | 3 | 2 |  |  |
| Diabetes, n (%) | Yes | 304 (22.7) | 208 (18.1) | 0.009 | 0.11 |
|  | No | 1033 (77.3) | 939 (81.9) |  |  |
| Missing, n |  | 6 | 12 |  |  |
| Smoking, n (%) | Yes | 517 (38.5) | 413 (35.8) | 0.182 | 0.05 |
|  | No | 826 (61.5) | 740 (64.2) |  |  |
| Missing, n |  | 0 | 6 |  |  |
| Drinking, n (%) | Yes | 463 (34.5) | 396 (34.4) | 0.947 | 0.00 |
|  | No | 879 (65.5) | 754 (65.6) |  |  |
| Missing, n |  | 1 | 9 |  |  |

**Supplementary Table S2：Baseline characteristics of participants in the CHARLS cross-sectional cohort according to sarcopenia status**

| **Variable** |  | **Sarcopenia** | **Non-sarcopenia** | **P value** |
| --- | --- | --- | --- | --- |
| Age |  | 70 (63, 76) | 61 (55, 67) | <0.001 |
| Sex, n (%) | Male | 68 (45.95) | 497 (41.59) | 0.311 |
|  | Female | 80 (54.05) | 698 (58.41) |  |
| Marital status, n (%) | Yes | 106 (71.62) | 1032 (86.36) | <0.001 |
|  | No | 42 (28.38) | 163 (13.64) |  |
| TyG |  | 8.48 (8.19,8.84) | 8.79 (8.36,9.24) | <0.001 |
| TyG-BMI |  | 163.53 (147.70,176.02) | 218.96 (190.59,247.84) | <0.001 |
| TyG-WWI |  | 95.03 (86.40,105.49) | 99.52 (92.04,108.28) | <0.001 |
| TyG-WC |  | 644.50 (591.64,697.23) | 785.27 (697.40,872.83) | <0.001 |
| TyG-WHtR |  | 4.16 (3.72,4.63) | 4.97 (4.42,5.55) | <0.001 |
| CTI |  | 8.55 (8.14,9.12) | 8.90 (8.36,9.55) | <0.001 |
| CMI |  | 0.82 (0.56,1.30) | 1.48 (0.84,2.57) | <0.001 |
| Non-hdlc |  | 130.09 (105.54,156.09) | 146.14 (119.27,170.68) | <0.001 |
| NHHR |  | 2.37 (1.91,3.01) | 3.19 (2.31,4.27) | <0.001 |
| RC |  | 17.78 (9.47,25.90) | 22.42 (13.53,36.53) | <0.001 |
| Hypertension, n (%) | Yes | 89 (60.14) | 778 (65.27) | 0.233 |
|  | No | 59 (39.86) | 414 (34.73) |  |
| Diabetes, n (%) | Yes | 23 (15.54) | 281 (23.63) | 0.029 |
|  | No | 125 (84.46) | 908 (76.37) |  |
| Smoking, n (%) | Yes | 65 (43.92) | 452 (37.82) | 0.151 |
|  | No | 83 (56.08) | 743 (62.18) |  |
| Drinking, n (%) | Yes | 55 (37.16) | 408 (34.17) | 0.466 |
|  | No | 93 (62.84) | 786 (65.83) |  |
| Abbreviations: TyG, triglyceride-glucose index; TyG-BMI, TyG-body mass index; TyG-WWI, TyG-weight-adjusted waist index; TyG-WC, TyG-waist circumference; TyG-WHtR, TyG-waist-to-height ratio; CTI, C-reactive protein–triglyceride-glucose index; CMI, cardiometabolic index; non-HDL-C, non-high-density lipoprotein cholesterol; NHHR, non-high-density lipoprotein cholesterol to high-density lipoprotein cholesterol ratio; RC, remnant cholesterol. | | | | |

**Supplementary Table S3：Baseline characteristics of the CHARLS longitudinal development cohort, training set, and internal validation set**

| **Variable** | **Category** | **Total**  **(N = 887)** | **Training set**  **(N = 620)** | **Internal validation set**  **(N = 267)** | **P value** |
| --- | --- | --- | --- | --- | --- |
| Age |  | 60 (54,66) | 60 (55,66) | 60 (54,66) | 0.885 |
| Sex, n (%) | Male | 372 (41.94) | 248 (40.00) | 124 (46.44) | 0.204 |
|  | Female | 515 (58.06) | 372 (60.00) | 143 (53.56) |  |
| Marital status, n (%) | Yes | 780 (87.94) | 547 (88.23) | 233 (87.27) | 0.922 |
|  | No | 107 (12.06) | 73 (11.77) | 34 (12.73) |  |
| TyG |  | 8.78 (8.36,9.23) | 8.79 (8.38,9.24) | 8.77 (8.35, 9.22) | 0.884 |
| TyG-BMI |  | 219 (192, 248) | 222 (194, 249) | 213 (189, 248) | 0.503 |
| TyG-WWI |  | 99 (92, 107) | 99 (92, 107) | 100 (92, 108) | 0.740 |
| TyG-WC |  | 784 (698, 868) | 787 (699, 865) | 768 (697, 872) | 0.930 |
| TyG-WHtR |  | 4.96 (4.41, 5.53) | 4.97 (4.44, 5.50) | 4.91 (4.35, 5.55) | 0.931 |
| CTI |  | 8.90 (8.34, 9.49) | 8.93 (8.34, 9.50) | 8.84 (8.34, 9.41) | 0.665 |
| CMI |  | 1.47 (0.84, 2.55) | 1.55 (0.89, 2.55) | 1.40 (0.78, 2.61) | 0.816 |
| Non-hdlc |  | 146 (119, 170) | 147 (121, 169) | 144 (118, 170) | 0.626 |
| NHHR |  | 3.15 (2.31, 4.24) | 3.20 (2.34, 4.23) | 3.03 (2.26, 4.30) | 0.689 |
| RC |  | 22 (14, 36) | 23 (14, 37) | 22 (13, 36) | 0.814 |
| Hypertension, n (%) | Yes | 568 (64.04) | 398 (64.19) | 170 (63.67) | 0.989 |
|  | No | 319 (35.96) | 222 (35.81) | 97 (36.33) |  |
| Diabetes, n (%) | Yes | 184 (20.74) | 122 (19.68) | 62 (23.22) | 0.490 |
|  | No | 703 (79.26) | 498 (80.32) | 205 (76.78) |  |
| Smoking, n (%) | Yes | 331 (37.32) | 217 (35.00) | 114 (42.70) | 0.094 |
|  | No | 556 (62.68) | 403 (65.00) | 153 (57.30) |  |
| Drinking, n (%) | Yes | 306 (34.50) | 200 (32.26) | 106 (39.70) | 0.102 |
|  | No | 581 (65.50) | 420 (67.74) | 161 (60.30) |  |

| Abbreviations: TyG, triglyceride-glucose index; TyG-BMI, TyG-body mass index; TyG-WWI, TyG-weight-adjusted waist index; TyG-WC, TyG-waist circumference; TyG-WHtR, TyG-waist-to-height ratio; CTI, C-reactive protein–triglyceride-glucose index; CMI, cardiometabolic index; non-HDL-C, non-high-density lipoprotein cholesterol; NHHR, non-high-density lipoprotein cholesterol to high-density lipoprotein cholesterol ratio; RC, remnant cholesterol. |
| --- |

**Supplementary Table S4：Baseline characteristics of the ELSA external validation cohort according to sarcopenia status**

| **Variable** |  | **Sarcopenia** | **Non-sarcopenia** | **P value** |
| --- | --- | --- | --- | --- |
| Age |  | 73 (68, 78) | 66 (60, 73) | <0.001 |
| Sex, n (%) | Male | 14 (56.00) | 434 (54.39) | 0.873 |
|  | Female | 11 (44.00) | 364 (45.61) |  |
| Marital status, n (%) | Yes | 10 (40.00) | 573 (71.80) | <0.001 |
|  | No | 15 (60.00) | 225 (28.20) |  |
| TyG |  | 8.16 (8.01, 8.57) | 8.59 (8.29, 8.94) | 0.001 |
| TyG-BMI |  | 188.00 (173.00, 200.00) | 240.00 (215.00, 273.00) | <0.001 |
| TyG-WWI |  | 91.00 (87.00, 95.00) | 95.00 (89.00, 102.00) | 0.023 |
| TyG-WC |  | 697.00 (628.00, 756.00) | 845.00 (754.00, 934.00) | <0.001 |
| TyG-WHtR |  | 4.27 (4.06, 4.61) | 5.05 (4.56, 5.60) | <0.001 |
| CTI |  | 8.64 (7.99, 9.19) | 8.90 (8.40, 9.40) | 0.171 |
| CMI |  | 0.77 (0.49, 1.04) | 1.31 (0.83, 2.00) | <0.001 |
| Non-hdlc |  | 128.00 (97.00, 151.00) | 135.00 (112.00, 162.00) | 0.131 |
| NHHR |  | 1.96 (1.42, 2.47) | 2.47 (1.93, 3.20) | 0.002 |
| RC |  | 15.00 (12.00, 27.00) | 23.00 (19.00, 35.00) | <0.001 |
| Hypertension, n (%) | Yes | 15 (60.00) | 430 (53.88) | 0.546 |
|  | No | 10 (40.00) | 368 (46.12) |  |
| Diabetes, n (%) | Yes | 2 (8.00) | 48 (6.02) | 0.660 |
|  | No | 23 (92.00) | 750 (93.98) |  |
| Smoking, n (%) | Yes | 9 (36.00) | 116 (14.54) | 0.008 |
|  | No | 16 (64.00) | 682 (85.46) |  |
| Drinking, n (%) | Yes | 18 (72.00) | 688 (86.22) | 0.072 |
|  | No | 7 (28.00) | 110 (13.78) |  |
| Abbreviations: TyG, triglyceride-glucose index; TyG-BMI, TyG-body mass index; TyG-WWI, TyG-weight-adjusted waist index; TyG-WC, TyG-waist circumference; TyG-WHtR, TyG-waist-to-height ratio; CTI, C-reactive protein–triglyceride-glucose index; CMI, cardiometabolic index; non-HDL-C, non-high-density lipoprotein cholesterol; NHHR, non-high-density lipoprotein cholesterol to high-density lipoprotein cholesterol ratio; RC, remnant cholesterol. | | | | |

**Supplementary Table S5：Baseline characteristics of the ELSA external validation cohort according to sarcopenia status**

| **Variable** |  | **Sarcopenia** | **Non-sarcopenia** | **P value** |
| --- | --- | --- | --- | --- |
| Age |  | 75 (68,81) | 65 (56,73) | <0.001 |
| Sex, n (%) | Male | 180 (40.63) | 944 (45.96) | 0.041 |
|  | Female | 263 (59.37) | 1110 (54.04) |  |
| Marital status, n (%) | Yes | 371 (83.75) | 1910 (92.99) | <0.001 |
|  | No | 72 (16.25) | 144 (7.01) |  |
| TyG |  | 8.49 (8.11 , 8.79) | 8.72 (8.35 , 9.04) | <0.001 |
| TyG-BMI |  | 176.41 (159.89 , 189.78) | 222.08 (201.85 , 244.51) | <0.001 |
| TyG-WWI |  | 76.96 (71.72 , 82.41) | 79.69 (74.66 , 84.59) | <0.001 |
| TyG-WC |  | 555.94 (507.51 , 591.68) | 645.13 (599.43 , 691.60) | <0.001 |
| TyG-WHtR |  | 3.52 (3.19 , 3.83) | 4.02 (3.71 , 4.34) | <0.001 |
| CTI |  | 8.42 (8.02, 8.96) | 8.60 (8.18, 9.27) | <0.001 |
| CMI |  | 0.81 (0.56 , 1.09) | 1.16 (0.81 , 1.66) | <0.001 |
| Non-hdlc |  | 121.50 (90.10 , 141.15) | 124.88 (97.84 , 146.87) | <0.001 |
| NHHR |  | 2.19 (1.69 , 2.64) | 2.46 (1.98 , 2.94) | <0.001 |
| RC |  | 20.88 (13.53 , 29.78) | 22.04 (14.31 , 31.63) | 0.026 |
| Hypertension, n (%) | Yes | 305 (68.85%) | 1,572 (76.53%) | <0.001 |
|  | No | 138 (31.15%) | 482 (23.47%) |  |
| Diabetes, n (%) | Yes | 102 (23.02%) | 518 (25.22%) | 0.332 |
|  | No | 341 (76.98%) | 1,536 (74.78%) |  |
| Abbreviations: TyG, triglyceride-glucose index; TyG-BMI, TyG-body mass index; TyG-WWI, TyG-weight-adjusted waist index; TyG-WC, TyG-waist circumference; TyG-WHtR, TyG-waist-to-height ratio; CTI, C-reactive protein–triglyceride-glucose index; CMI, cardiometabolic index; non-HDL-C, non-high-density lipoprotein cholesterol; NHHR, non-high-density lipoprotein cholesterol to high-density lipoprotein cholesterol ratio; RC, remnant cholesterol. | | | | |

**Supplementary Table S6: AUCs of composite biomarkers across three adjustment models in the CHARLS cross-sectional cohort**

| **Biomarker** | **CHARLS** | | |
| --- | --- | --- | --- |
|  | **Model 1**  **AUC(95%CI)** | **Model 2**  **AUC(95%CI)** | **Model 3**  **AUC(95%CI)** |
| TyG | 0.640(0.597,0.683) | 0.791(0.755,0.827) | 0.797(0.762,0.833) |
| TyG-BMI | 0.902(0.880,0.923) | 0.937(0.921,0.953) | 0.938(0.922,0.954) |
| TyG-WWI | 0.588(0.538,0.639) | 0.785(0.747,0.823) | 0.793(0.757,0.829) |
| TyG-WC | 0.819(0.787,0.851) | 0.878(0.853,0.903) | 0.878(0.853,0.903) |
| TyG-WHtR | 0.761(0.722,0.801) | 0.858(0.830,0.886) | 0.858(0.830,0.886) |
| CTI | 0.614(0.569,0.659) | 0.791(0.754,0.827) | 0.797(0.762,0.832) |
| CMI | 0.706(0.665,0.748) | 0.814(0.779,0.849) | 0.821(0.787,0.855) |
| Non-hdlc | 0.598(0.549,0.647) | 0.778(0.740,0.816) | 0.789(0.753,0.826) |
| NHHR | 0.668(0.623,0.713) | 0.801(0.766,0.837) | 0.810(0.775,0.844) |
| RC | 0.617(0.570,0.663) | 0.783(0.746,0.821) | 0.793(0.757,0.829) |
| Abbreviations: AUC, area under the curve; CI, confidence interval.  Model 1 : was unadjusted.  Model 2 : was adjusted for age, sex, and marital status in the overall analysis.  Model 3 : was further adjusted for smoking, alcohol consumption, hypertension, and diabetes. | | | |

**Supplementary Table S7：Performance comparison of the nine machine learning algorithms across the development and validation datasets**

| **Classifier** | **Accuracy** | **Specificity** | **AUC** | **NPV** |
| --- | --- | --- | --- | --- |
| **Training set** |  |  |  |  |
| Logistic | 0.942 | 0.995 | 0.898 | 0.946 |
| RF | 0.940 | 1.000 | 0.917 | 0.940 |
| Decision Tree | 0.948 | 0.995 | 0.909 | 0.952 |
| LGBM | 1.000 | 1.000 | 1.000 | 1.000 |
| CatBoost | 0.966 | 1.000 | 0.982 | 0.965 |
| SVM | 0.940 | 1.000 | 0.767 | 0.940 |
| MLP | 0.940 | 1.000 | 0.889 | 0.940 |
| NB | 0.902 | 0.928 | 0.878 | 0.966 |
| KNNC | 0.945 | 0.993 | 0.941 | 0.951 |
| mean_scores | 0.947 | 0.990 | 0.909 | 0.955 |
| **Internal validation set** |  |  |  |  |
| Logistic | 0.899 | 0.987 | 0.884 | 0.907 |
| RF | 0.888 | 1.000 | 0.877 | 0.888 |
| Decision Tree | 0.888 | 0.970 | 0.824 | 0.909 |
| LGBM | 0.899 | 0.970 | 0.870 | 0.920 |
| CatBoost | 0.895 | 0.996 | 0.907 | 0.897 |
| SVM | 0.888 | 1.000 | 0.653 | 0.888 |
| MLP | 0.895 | 1.000 | 0.872 | 0.894 |
| NB | 0.888 | 0.937 | 0.875 | 0.937 |
| KNNC | 0.884 | 0.987 | 0.786 | 0.893 |
| mean_scores | 0.891 | 0.983 | 0.839 | 0.903 |
| **ELSA external validation cohort** |  |  |  |  |
| Logistic | 0.962 | 0.987 | 0.949 | 0.974 |
| RF | 0.970 | 1.000 | 0.905 | 0.970 |
| Decision Tree | 0.959 | 0.984 | 0.762 | 0.974 |
| LGBM | 0.955 | 0.979 | 0.902 | 0.975 |
| CatBoost | 0.965 | 0.994 | 0.891 | 0.971 |
| SVM | 0.970 | 1.000 | 0.754 | 0.970 |
| MLP | 0.970 | 0.999 | 0.940 | 0.971 |
| NB | 0.922 | 0.930 | 0.943 | 0.989 |
| KNNC | 0.966 | 0.992 | 0.761 | 0.973 |
| mean_scores | 0.960 | 0.985 | 0.867 | 0.974 |
| **Clinical external validation cohort** |  |  |  |  |
| Logistic | 0.871 | 0.976 | 0.909 | 0.880 |
| RF | 0.823 | 1.000 | 0.903 | 0.823 |
| Decision Tree | 0.841 | 0.983 | 0.836 | 0.848 |
| LGBM | 0.848 | 0.975 | 0.886 | 0.859 |
| CatBoost | 0.830 | 0.987 | 0.900 | 0.836 |
| SVM | 0.823 | 1.000 | 0.580 | 0.823 |
| MLP | 0.842 | 0.997 | 0.910 | 0.841 |
| NB | 0.839 | 0.863 | 0.888 | 0.936 |
| KNNC | 0.843 | 0.988 | 0.810 | 0.846 |
| mean_scores | 0.840 | 0.974 | 0.847 | 0.852 |
| Abbreviations: RF, Random Forest; LGBM, Light Gradient Boosting Machine; SVM, Support Vector Machine; MLP, Multi-Layer Perceptron; NB, Naive Bayes; KNNC, K-Nearest Neighbors Classifier; AUC, area under the curve; NPV, negative predictive value.  Performance metrics include accuracy, specificity, AUC, and NPV. | | | | |

**Supplementary Table S8：Detailed performance metrics of the final CatBoost model across the development and validation datasets**

| **Metric** | **CatBoost** | | | |
| --- | --- | --- | --- | --- |
|  | **CHARLS** | **CHARLS** | **ELSA** | **Clinical Cohort** |
|  | **Training set** | **Internal validation set** | **External validation set** | **External validation set** |
| Accuracy | 0.966 | 0.895 | 0.965 | 0.830 |
| Prevalence | 0.060 | 0.112 | 0.030 | 0.177 |
| F1-Score | 0.604 | 0.176 | 0.065 | 0.175 |
| AUC | 0.982 | 0.907 | 0.891 | 0.900 |
| Specificity | 1.000 | 0.996 | 0.994 | 0.987 |
| FNR | 0.568 | 0.900 | 0.960 | 0.898 |
| FPR | 0.000 | 0.004 | 0.006 | 0.013 |
| PPV | 1.000 | 0.750 | 0.167 | 0.634 |
| NPV | 0.965 | 0.897 | 0.971 | 0.836 |
| Abbreviations:CatBoost, Categorical Boosting  Performance metrics: AUC, Area Under the receiver operating characteristic curve; FNR,False Negative Rate; FPR,False Positive Rate; PPV,Positive Predictive Value; NPV,Negative Predictive Value | | | | |

**Supplementary Table S9：Ten-fold cross-validation performance of the CatBoost model**

| **Fold** | **Accuracy** | **AUC** | **Specificity** | **NPV** |
| --- | --- | --- | --- | --- |
| Fold 1 | 0.968 | 0.918 | 0.984 | 0.984 |
| Fold 2 | 0.952 | 0.892 | 0.983 | 0.967 |
| Fold 3 | 0.887 | 0.829 | 1.000 | 0.887 |
| Fold 4 | 0.919 | 0.858 | 0.983 | 0.934 |
| Fold 5 | 0.903 | 0.916 | 0.982 | 0.918 |
| Fold 6 | 0.952 | 0.774 | 1.000 | 0.952 |
| Fold 7 | 0.952 | 0.949 | 1.000 | 0.952 |
| Fold 8 | 0.935 | 0.931 | 1.000 | 0.935 |
| Fold 9 | 0.919 | 0.804 | 1.000 | 0.919 |
| Fold 10 | 0.952 | 0.904 | 1.000 | 0.952 |
| Mean | 0.934 | 0.877 | 0.993 | 0.940 |
| Abbreviations: CatBoost, Categorical Boosting;  Performance metrics: Accuracy, overall classification correctness; Specificity, true negative rate; AUC, Area Under the receiver operating characteristic curve; NPV, Negative Predictive Value | | | | |

**Supplementary Table S10：Pairwise DeLong comparisons of AUCs between TyG-BMI and other composite biomarkers**

| **Comparison** | **AUC difference (95% CI)** | **P value** |
| --- | --- | --- |
| TyG-BMI vs TyG | 0.134 (0.174, 0.094) | 0.0007 |
| TyG-BMI vs TyG-WWI | 0.103 (0.135, 0.071) | 0.0043 |
| TyG-BMI vs TyG-WC | 0.070 (0.094, 0.046) | 0.0066 |
| TyG-BMI vs TyG-WHtR | 0.051 (0.066, 0.036) | 0.0071 |
| TyG-BMI vs CTI | 0.119 (0.148, 0.089) | 0.0002 |
| TyG-BMI vs CMI | 0.115 (0.150, 0.079) | 0.0006 |
| TyG-BMI vs Non-hdlc | 0.162 (0.215, 0.109) | 0.0005 |
| TyG-BMI vs NHHR | 0.172 (0.232, 0.112) | 0.0004 |
| TyG-BMI vs RC | 0.211 (0.268, 0.154) | <0.0001 |
| Footnote:  AUC differences were calculated as AUC(TyG-BMI) − AUC(comparator). P values were obtained using DeLong’s test for paired ROC curves.  Abbreviations: AUC, area under the curve; CI, confidence interval; TyG, triglyceride-glucose index; TyG-BMI, TyG-body mass index; TyG-WWI, TyG-weight-adjusted waist index; TyG-WC, TyG-waist circumference; TyG-WHtR, TyG-waist-to-height ratio; CTI, C-reactive protein–triglyceride-glucose index; CMI, cardiometabolic index; non-HDL-C, non-high-density lipoprotein cholesterol; NHHR, non-high-density lipoprotein cholesterol to high-density lipoprotein cholesterol ratio; RC, remnant cholesterol. | | |

**Supplementary Table S11：NRI and IDI for TyG-BMI compared with other composite biomarkers**

|  | Tyg | Tyg-WWI | TygWC | TyG-WHtR | CTI | CMI | Non-HDLC | NHHR | RC |
| --- | --- | --- | --- | --- | --- | --- | --- | --- | --- |
| **NRI** | 1.036  (0.703,1.370) | 1.069  (0.748,1.390) | 0.868  (0.522,1.215) | 0.851  (0.513,1.188) | 0.844  (0.489,1.199) | 1.079  (0.746,1.411) | 1.019  (0.686,1.353) | 1.062  (0.729,1.394) | 1.269  (1.003,1.535) |
| **P** | <0.001 | <0.001 | <0.001 | <0.001 | <0.001 | <0.001 | <0.001 | <0.001 | <0.001 |
| **IDI** | 0.139  (0.075,0.203) | 0.115  (0.062,0.167) | 0.070  (0.008,0.132) | 0.063  (0.004,0.123) | 0.122  (0.063,0.182) | 0.130  (0.076,0.184) | 0.125  (0.061,0.188) | 0.134  (0.071,0.197) | 0.155  (0.092,0.219) |
| **P** | <0.001 | <0.001 | 0.028 | 0.036 | 0.001 | <0.001 | 0.001 | <0.001 | <0.001 |
| Footnote:  Values are presented as estimate (95% CI). Positive NRI or IDI values indicate improved classification or discrimination of the TyG-BMI model relative to the comparator model. P values were calculated using asymptotic Wald tests.  Abbreviations: NRI, net reclassification improvement; IDI, integrated discrimination improvement; CI, confidence interval; TyG, triglyceride-glucose index; TyG-BMI, TyG-body mass index; TyG-WWI, TyG-weight-adjusted waist index; TyG-WC, TyG-waist circumference; TyG-WHtR, TyG-waist-to-height ratio; CTI, C-reactive protein–triglyceride-glucose index; CMI, cardiometabolic index; non-HDL-C, non-high-density lipoprotein cholesterol; NHHR, non-high-density lipoprotein cholesterol to high-density lipoprotein cholesterol ratio; RC, remnant cholesterol. | | | | | | | | | |

**Supplementary Table S12：Associations of selected risk factors with the CVD-SRS in the training and internal validation sets**

|  | **Training set** | | | | **Internal validation set** | | | |
| --- | --- | --- | --- | --- | --- | --- | --- | --- |
| **Variable Name** | **β(95%CI)** | **Std Error** | **T value** | **P value** | **β(95%CI)** | **Std Error** | **T value** | **P value** |
| Age [45,50] | -0.264  (-0.493, -0.036) | 0.116 | -2.275 | 0.023 | -0.230  (-0.549, 0.089) | 0.162 | -1.420 | 0.157 |
| Age [50,70] | 0.2152  (-0.030, 0.460) | 0.125 | 1.725 | 0.085 | 0.118  (-0.225, 0.461) | 0.174 | 0.677 | 0.499 |
| Age > 70 | 0.767  (0.456, 1.078) | 0.158 | 4.844 | <0.001 | 1.323  (0.852, 1.793) | 0.239 | 5.537 | <0.001 |
| Sex[Male] | 0.266  (0.106, 0.426) | 0.081 | 3.267 | 0.001 | 0.367  (0.129, 0.605) | 0.121 | 3.037 | 0.003 |
| Sex[Female] | -0.106  (-0.207, -0.005) | 0.051 | -2.066 | 0.039 | -0.170  (-0.333, -0.008) | 0.082 | -2.069 | 0.040 |
| Marry[Yes] | -0.596  (-0.836, -0.355) | 0.122 | -4.868 | <0.001 | -0.351  (-0.710, 0.009) | 0.183 | -1.920 | 0.056 |
| Marry[No] | 0.526  (0.300, 0.751) | 0.115 | 4.573 | <0.001 | 0.306  (-0.030, 0.642) | 0.171 | 1.794 | 0.074 |
| Footnote:  β coefficients are presented with 95% confidence intervals. Estimates were derived from regression analyses of the CVD-SRS in the training and internal validation sets.  Abbreviations: CI, confidence interval; CVD-SRS, Cardiovascular Disease–Sarcopenia Risk Score. | | | | | | | | |

**Supplementary Figure S1：Receiver operating characteristic curves of composite biomarkers across three adjustment models in the CHARLS cross-sectional cohort**


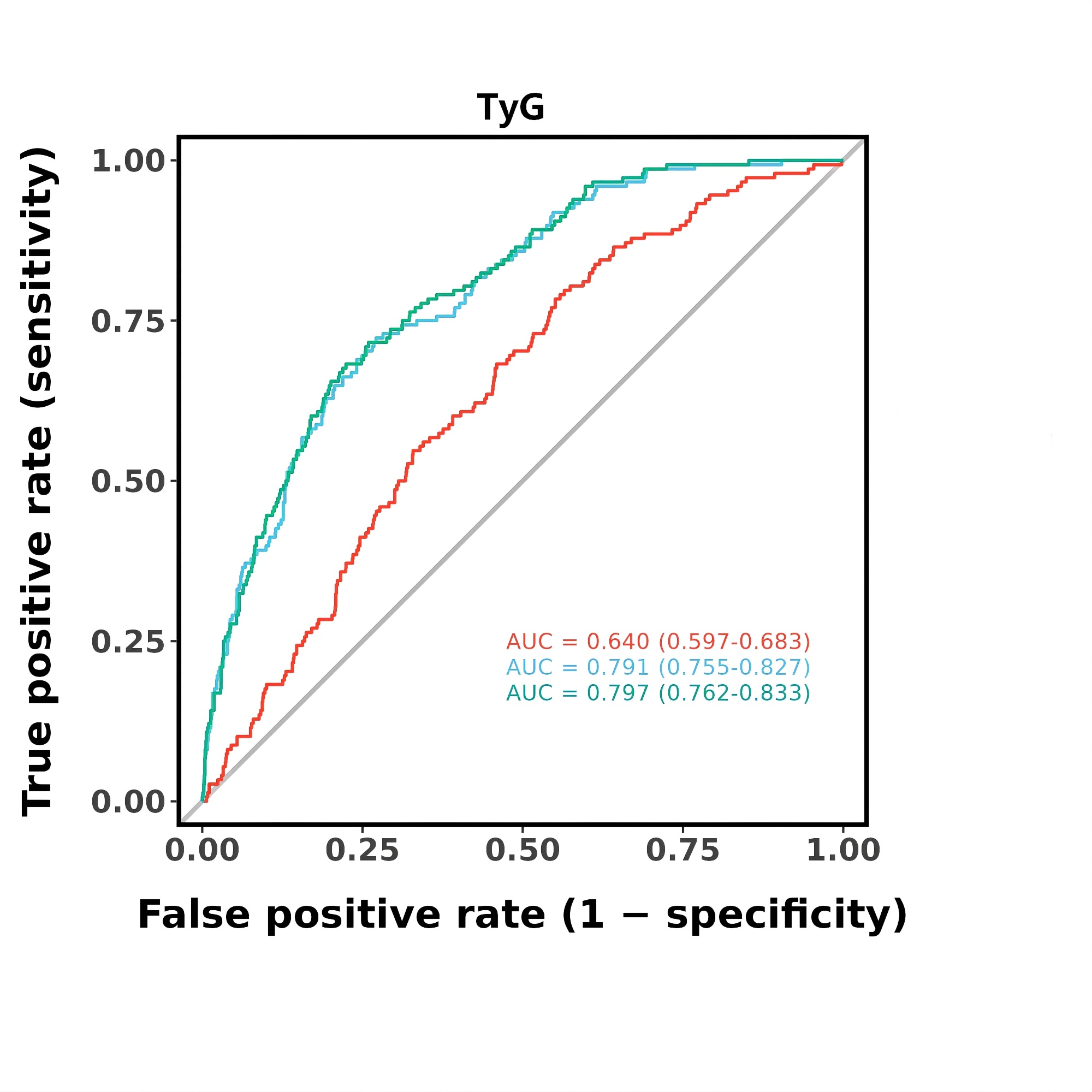

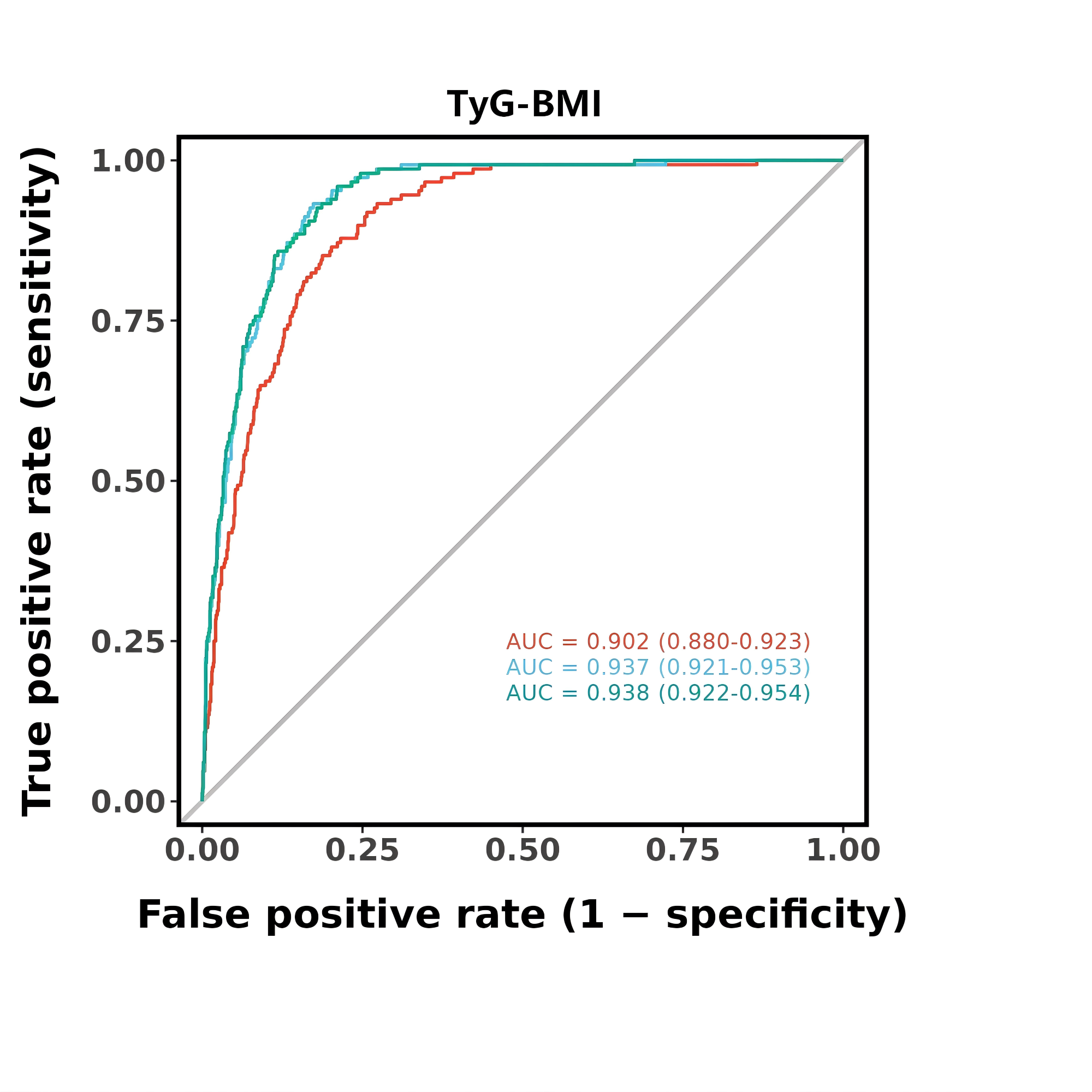

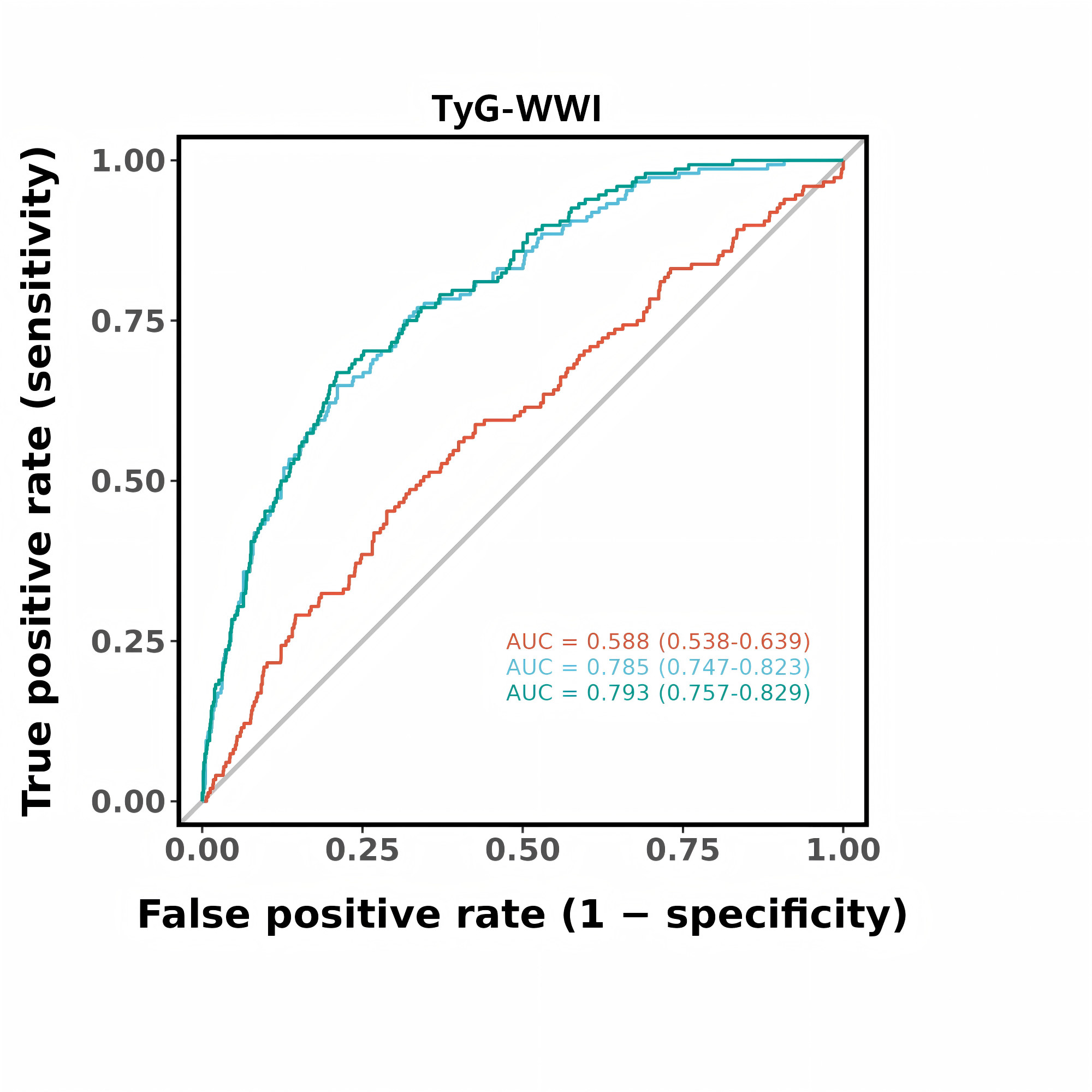

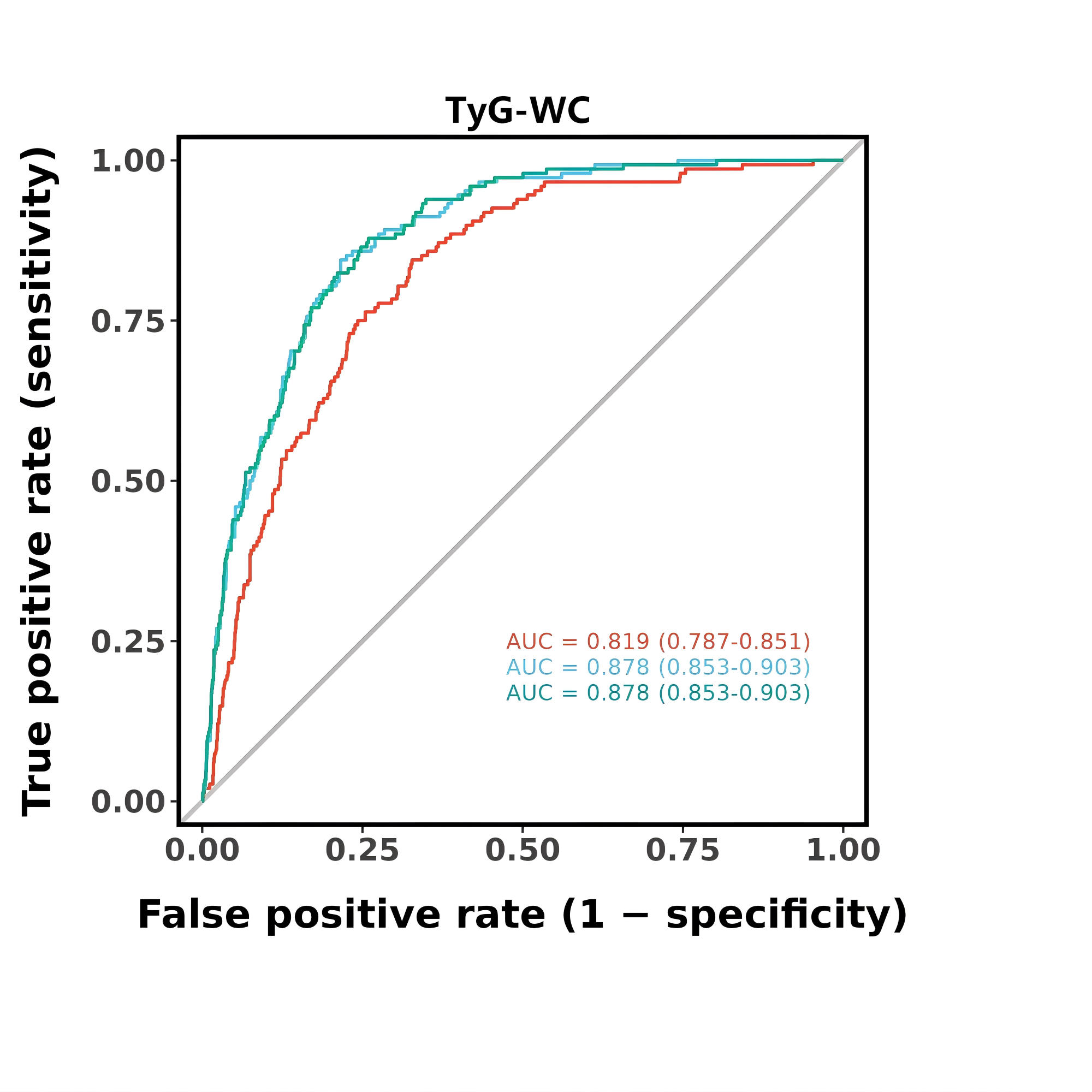

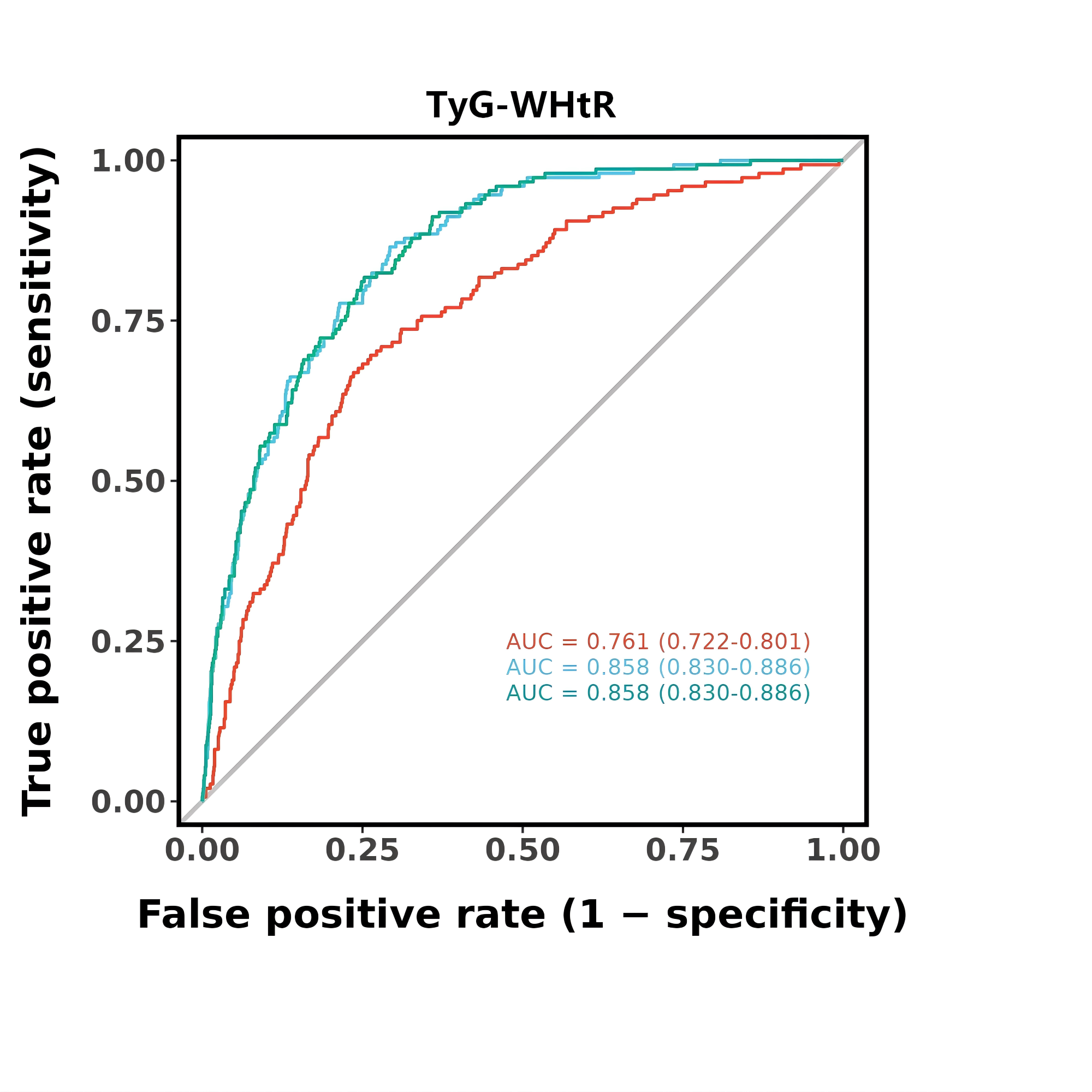

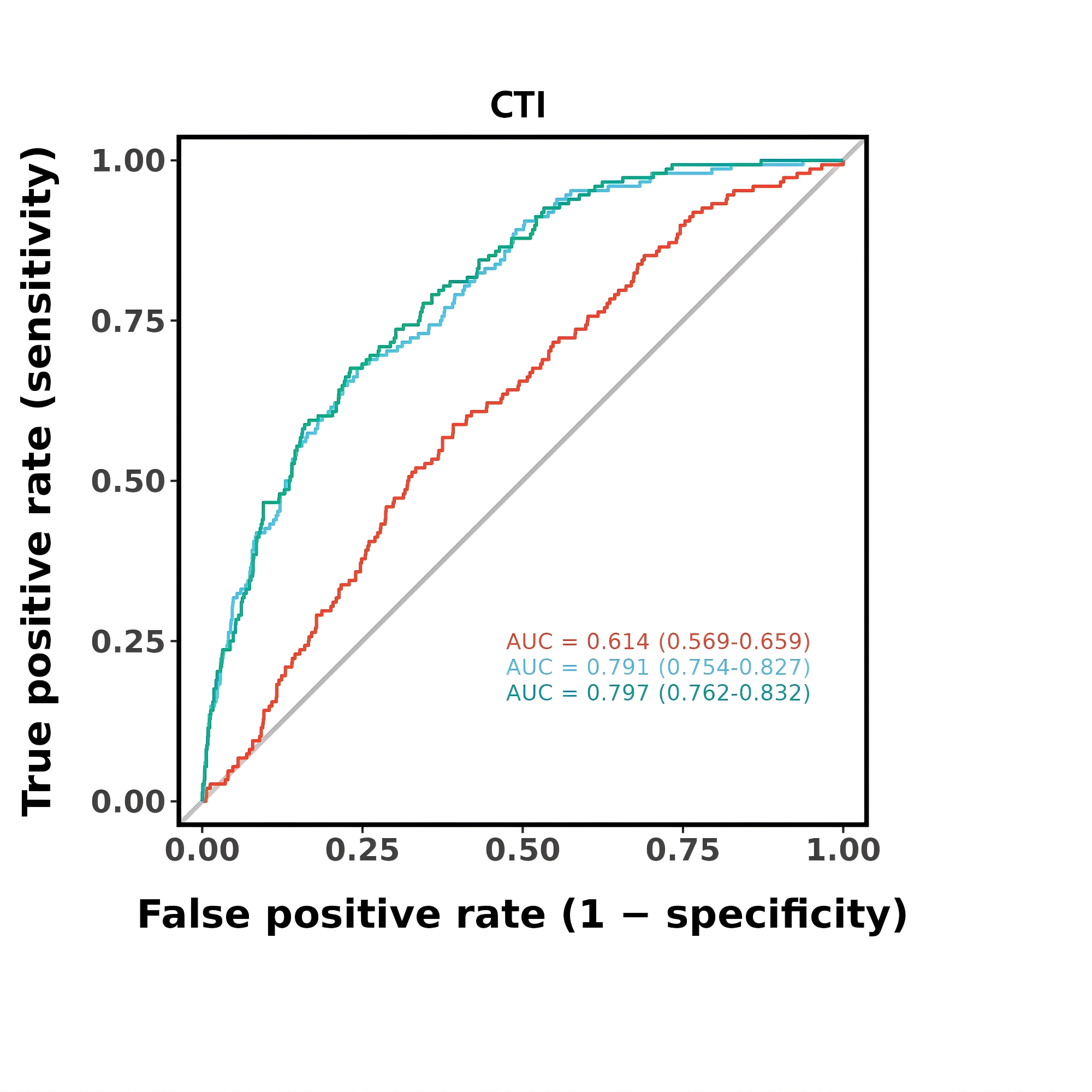

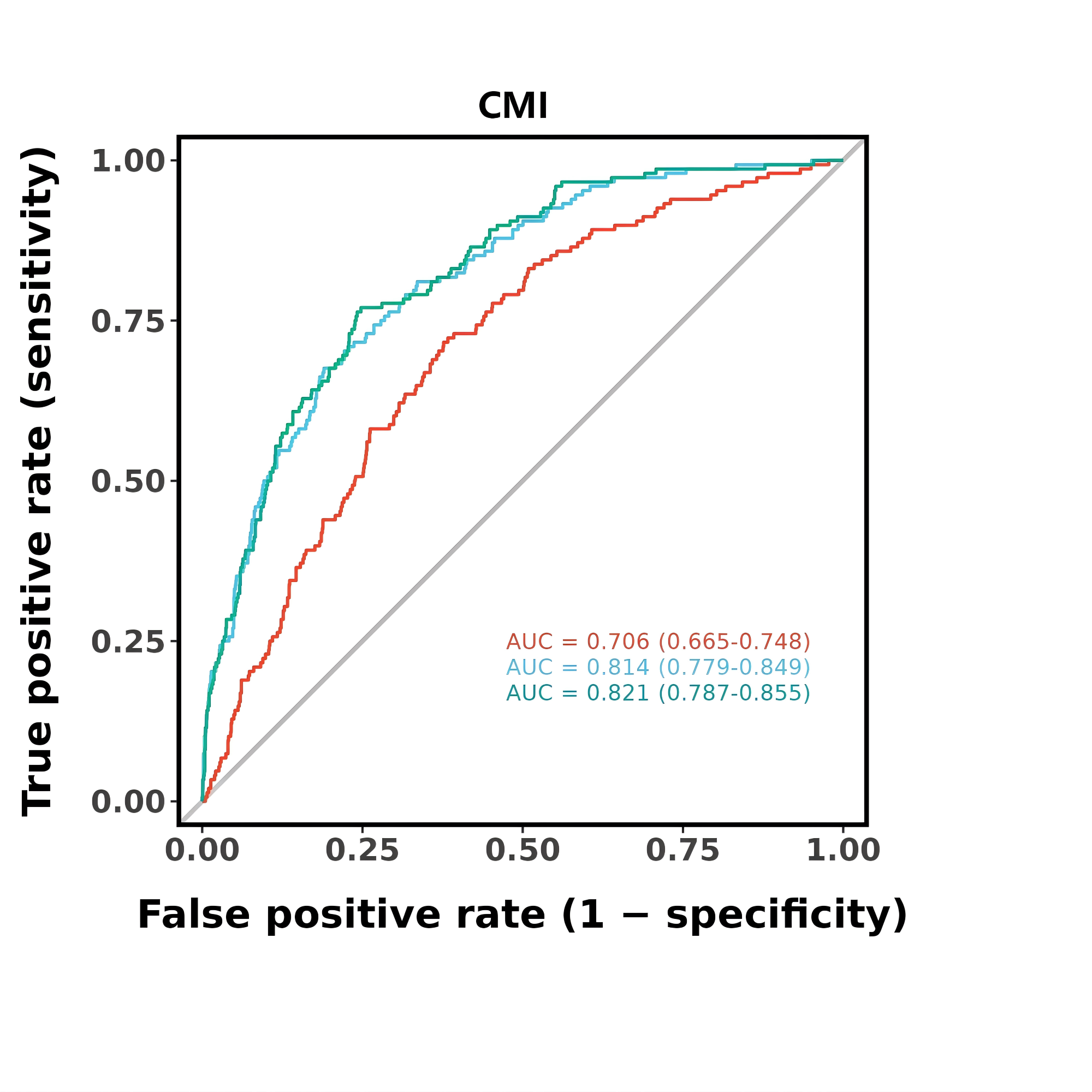

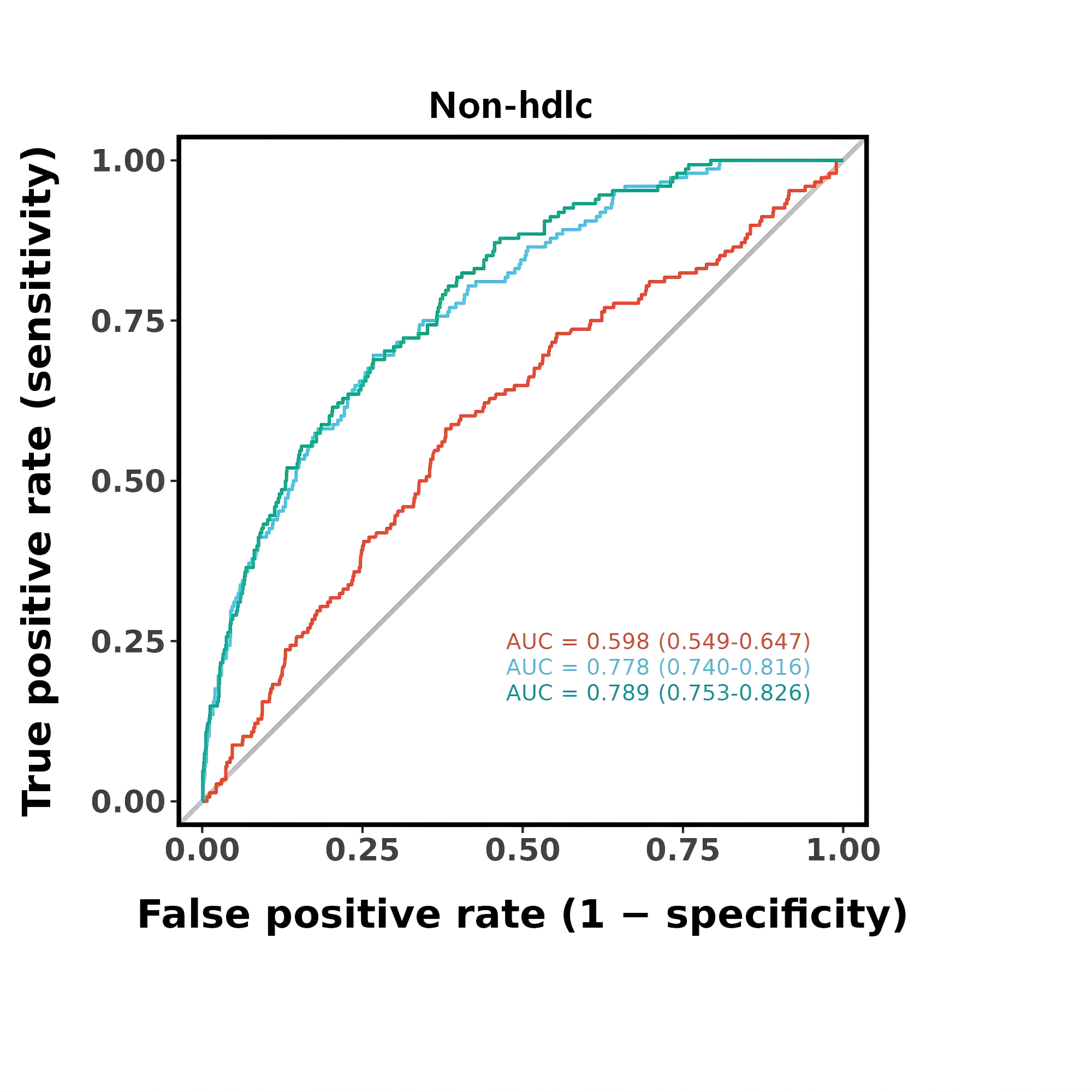

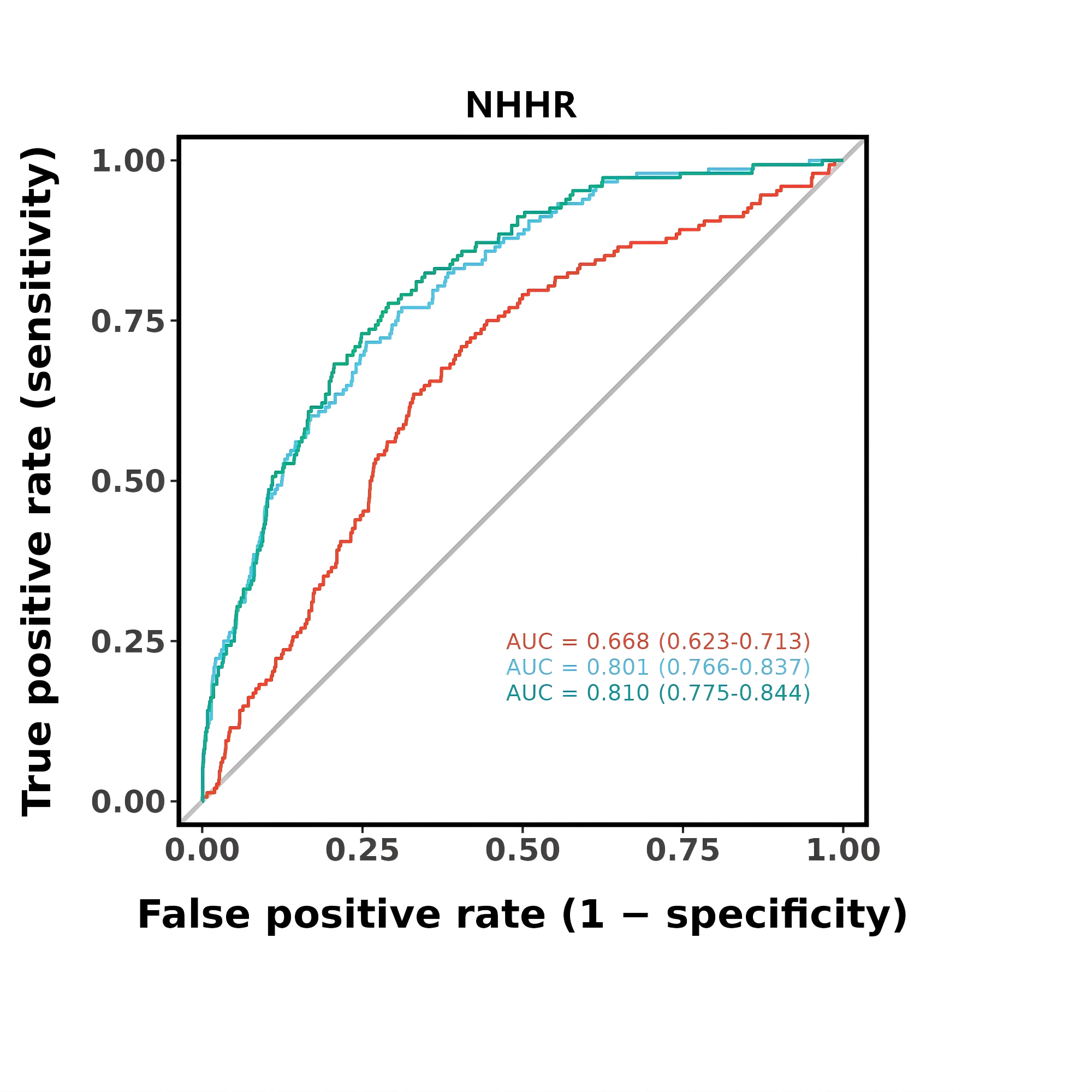

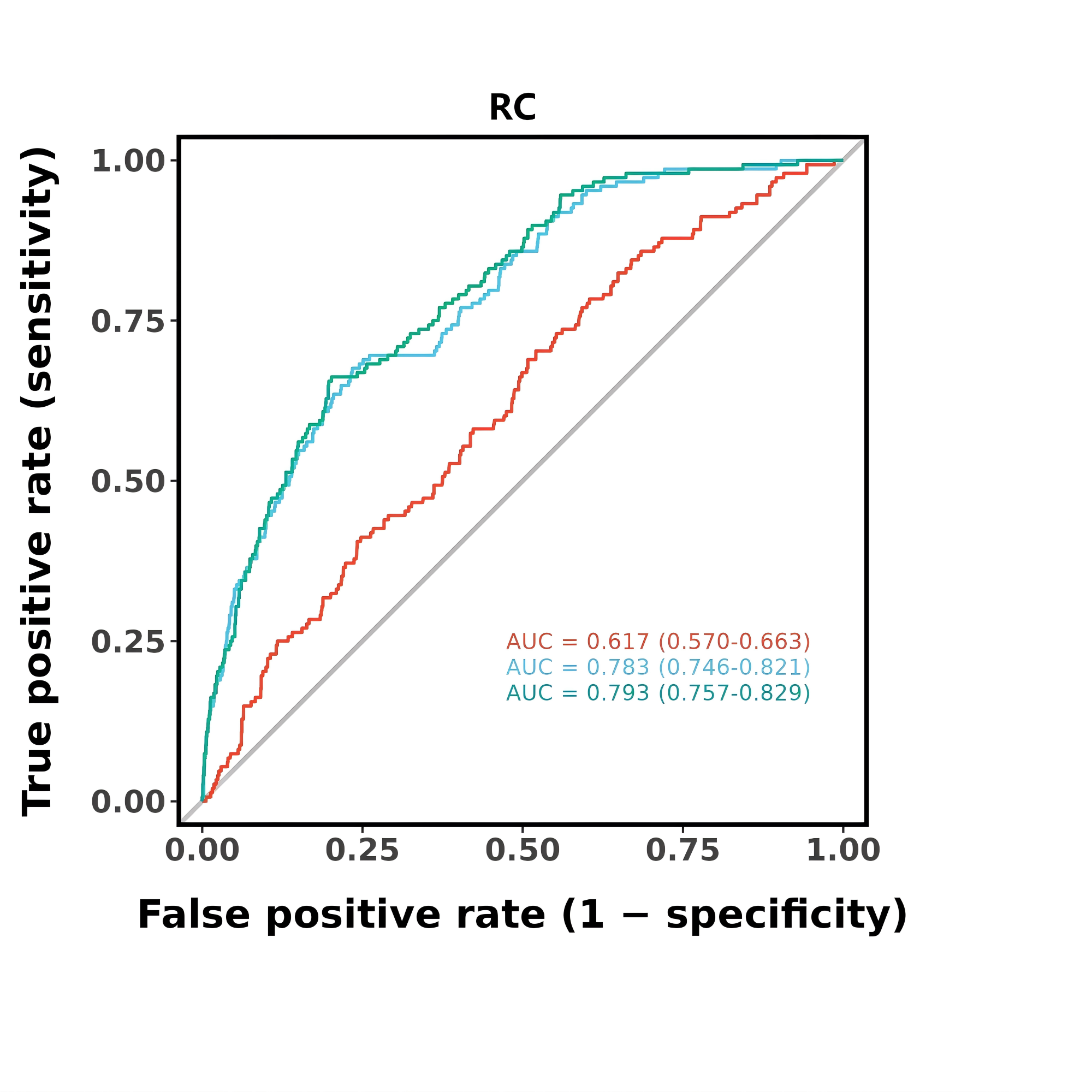

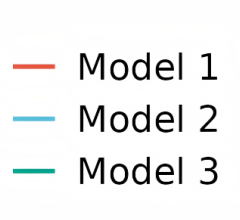


Legend:

Red lines indicate Model 1, blue lines indicate Model 2, and green lines indicate Model 3.

Model 1 was unadjusted.

Model 2 was adjusted for age, sex, and marital status.

Model 3 was further adjusted for smoking, alcohol consumption, hypertension, and diabetes.

AUC values are shown within each panel.

**Supplementary Figure S2：Additional evaluation of the final CatBoost model in the training and internal validation sets**

**Train**


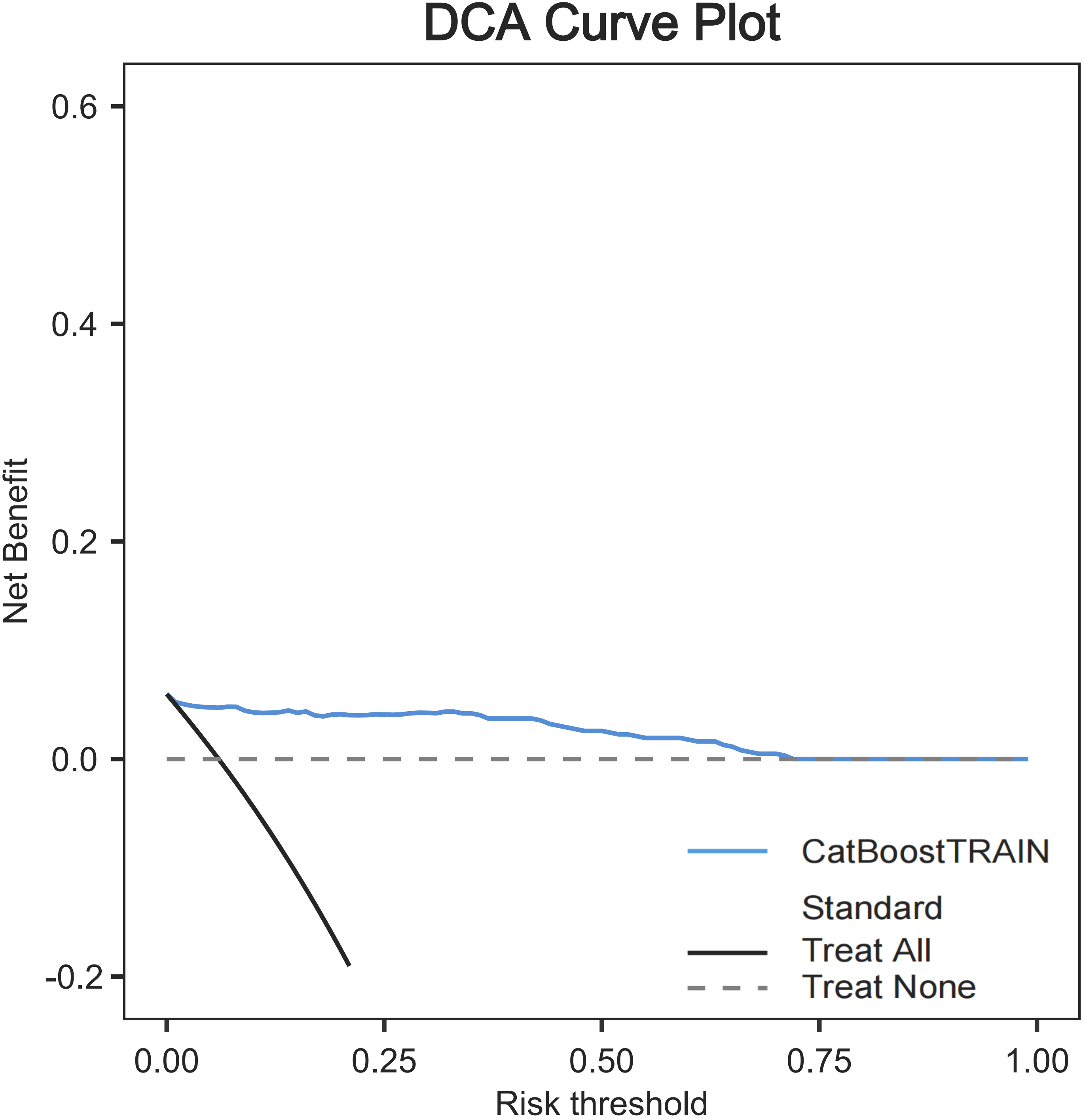

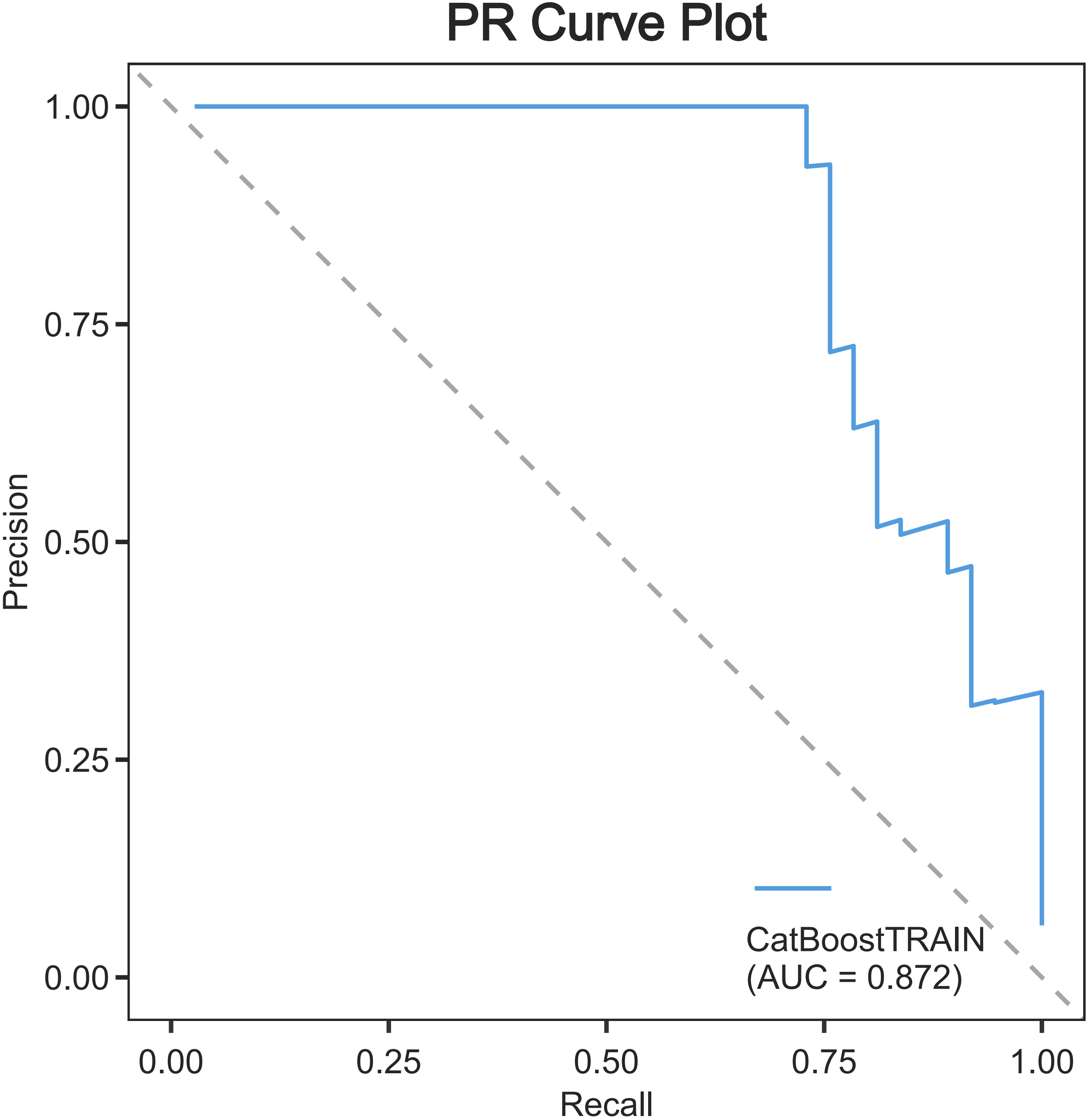

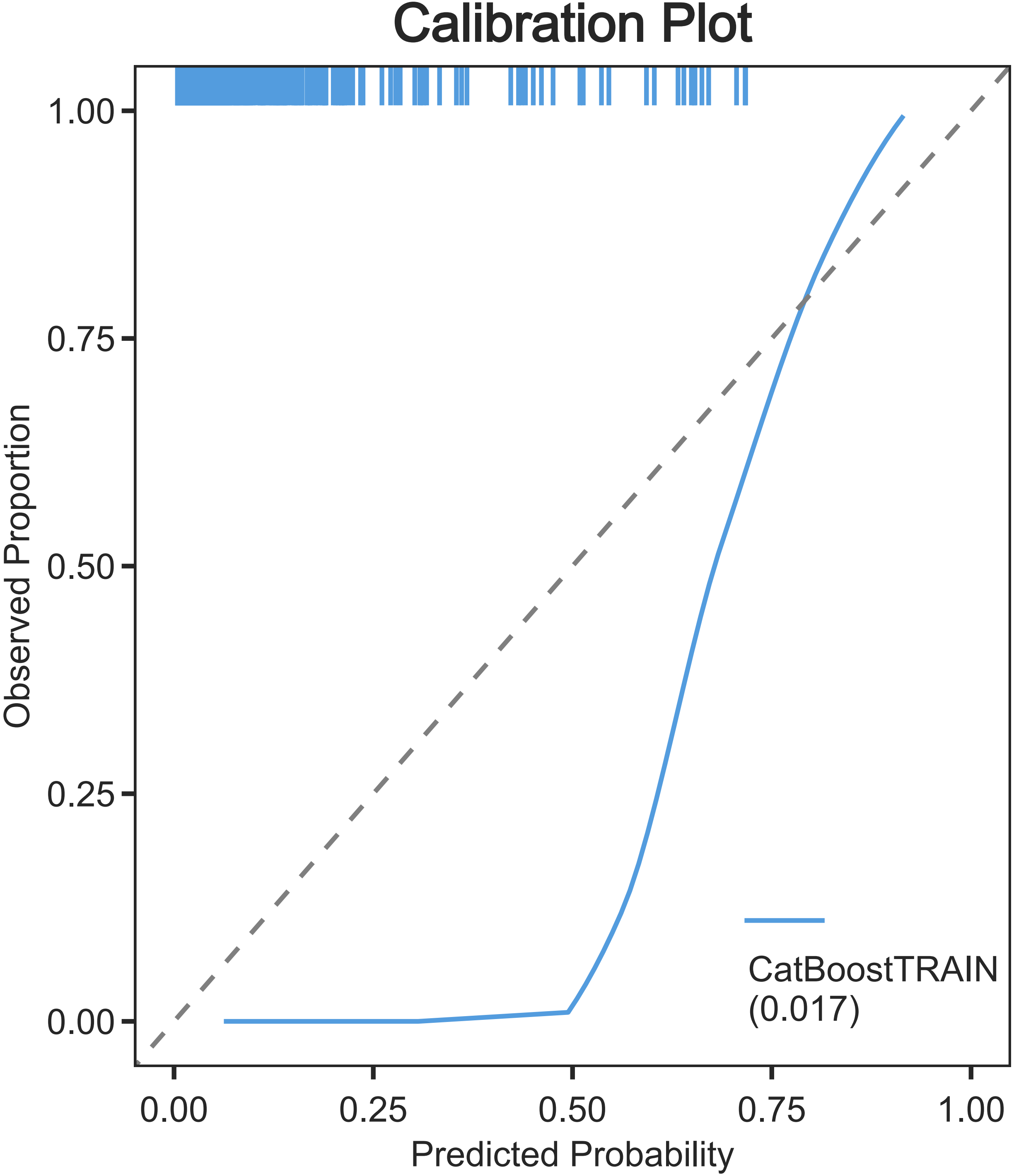


**Internal Test**

**
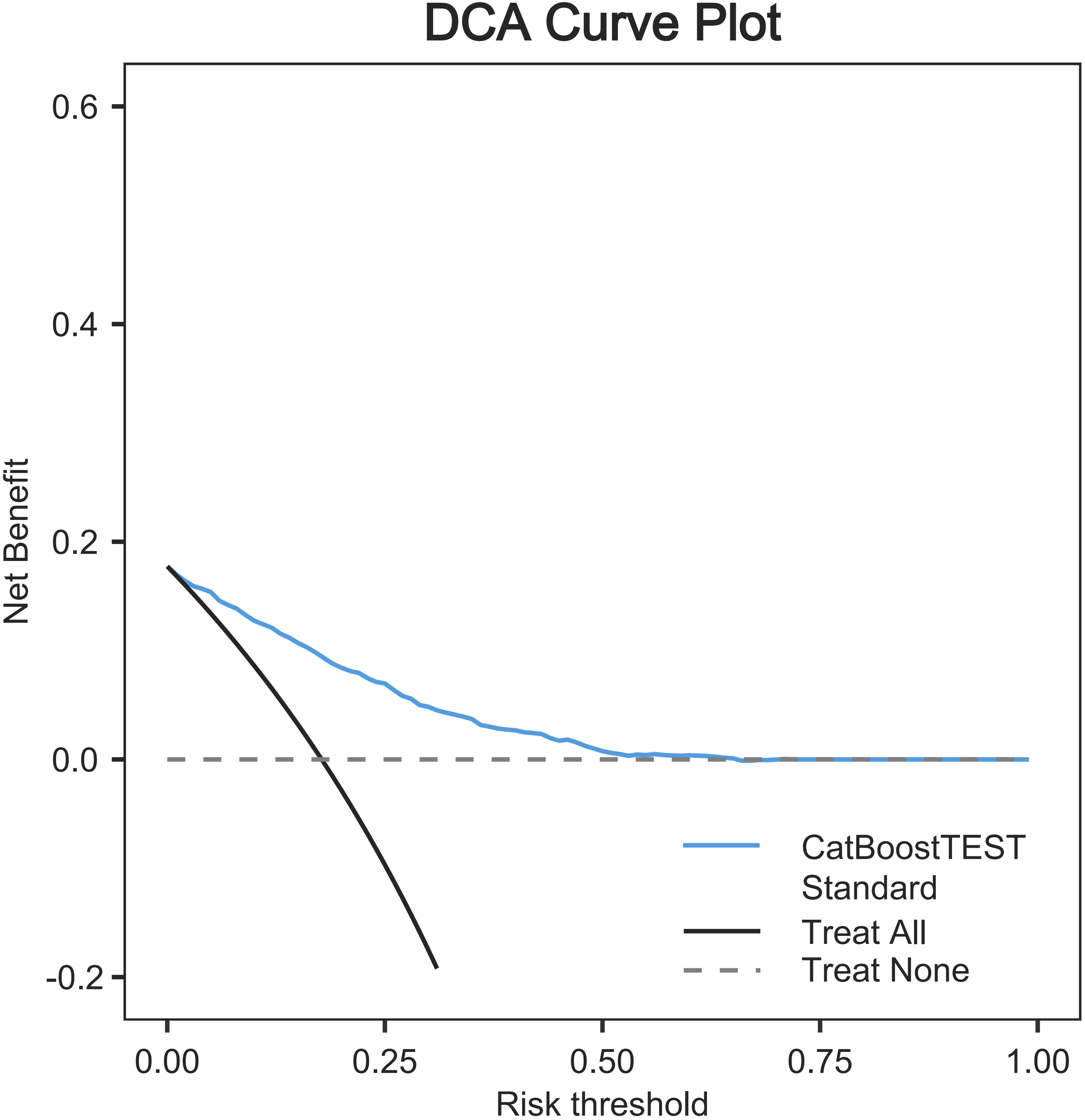

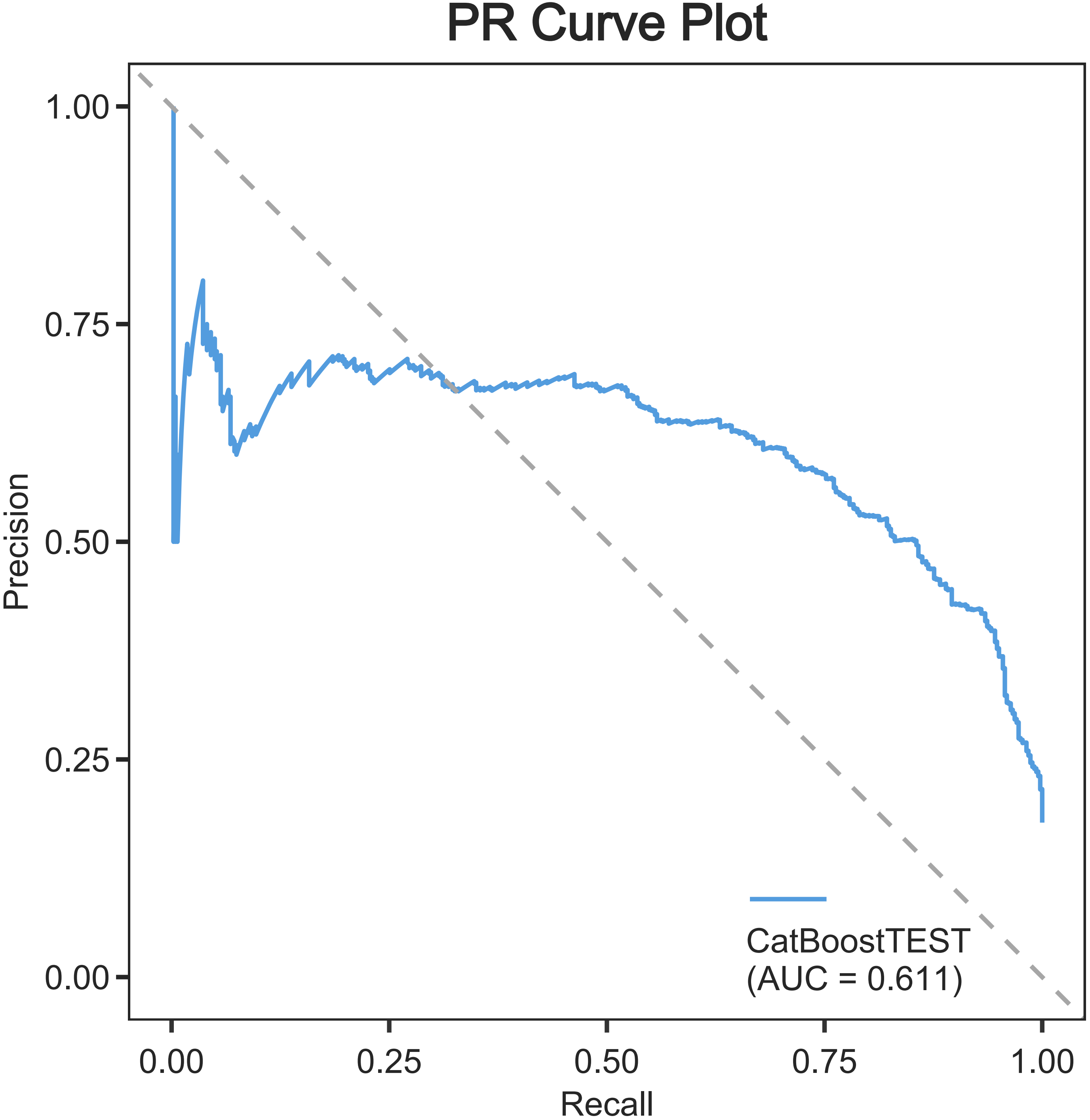

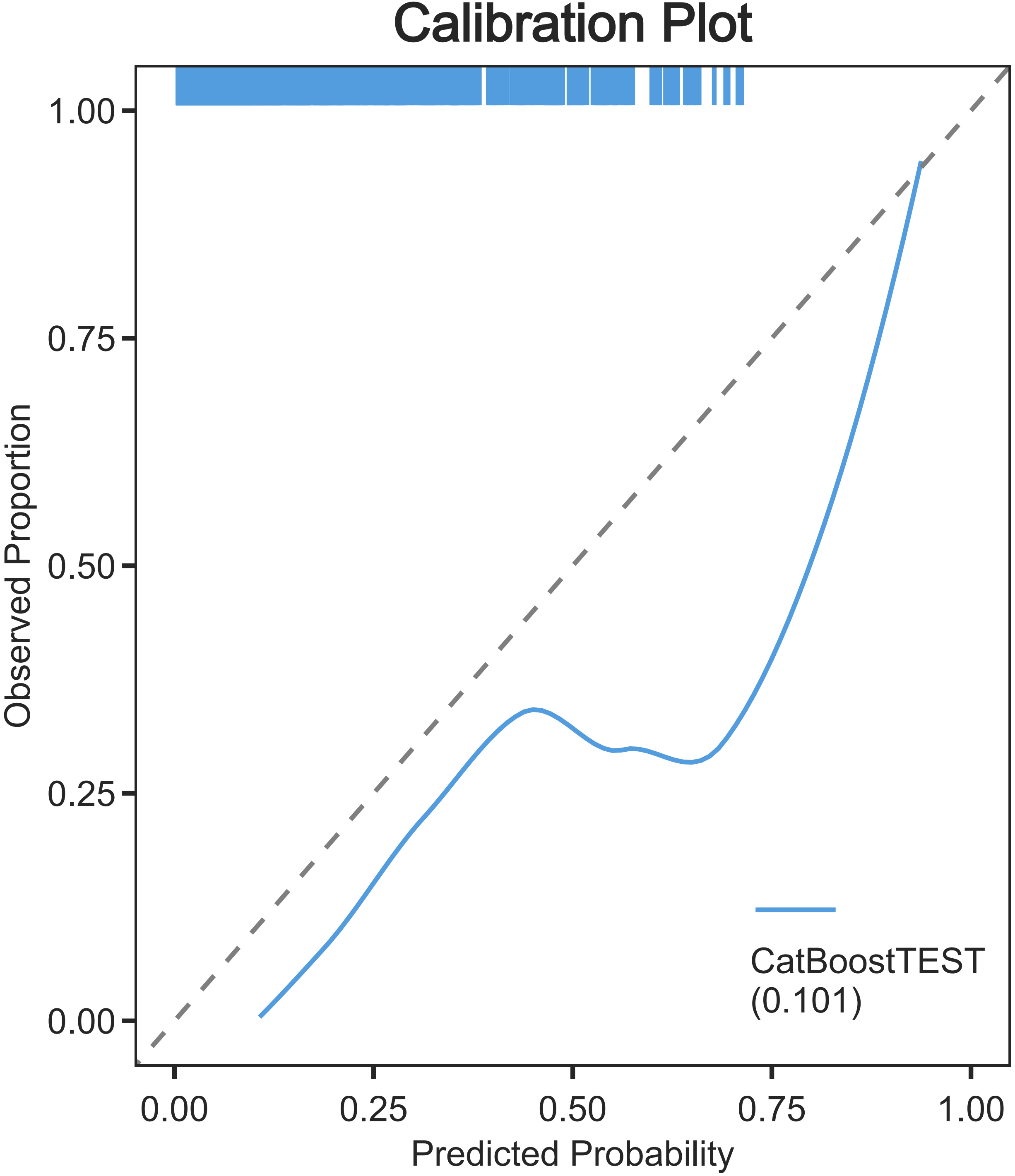
**

Legend:

Abbreviations: DCA, decision curve analysis; PR, precision-recall.

**supplementary Figure S3：SHAP-based interpretation of the final CatBoost model in the training and internal validation sets**

**A Train D Internal Test**


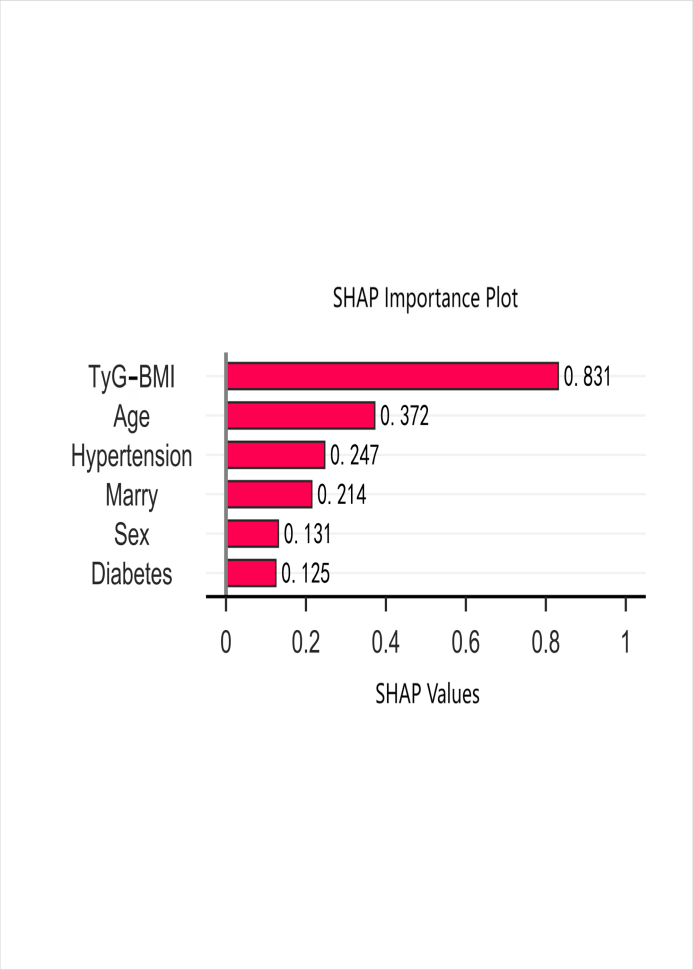

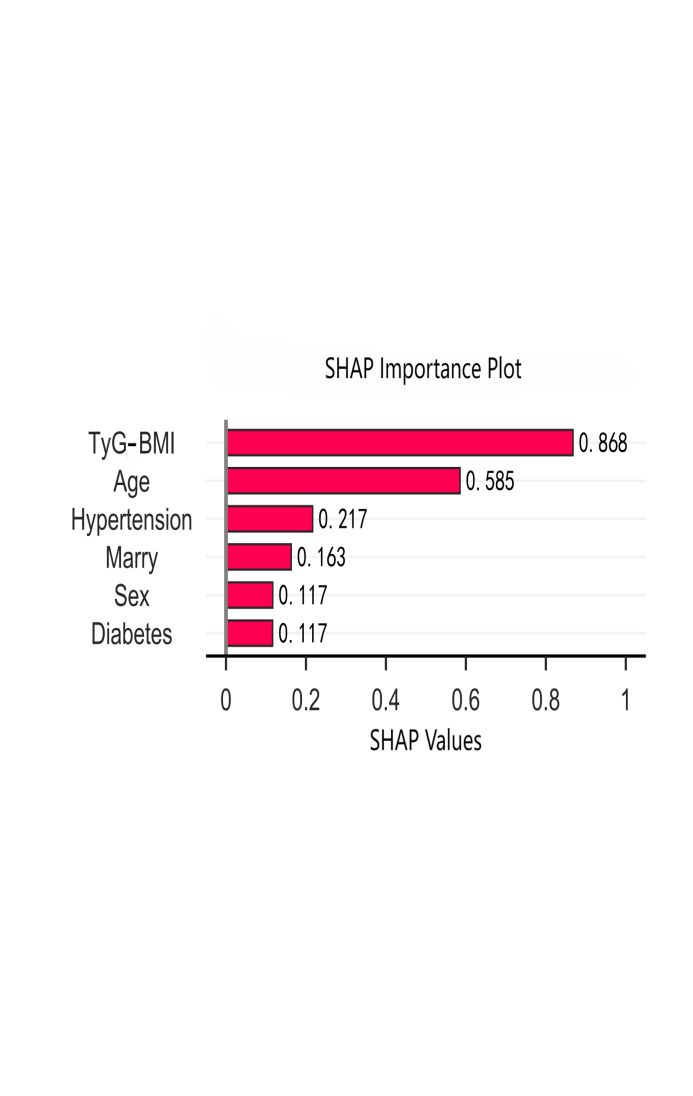


**B Train E Internal Test**

**

**
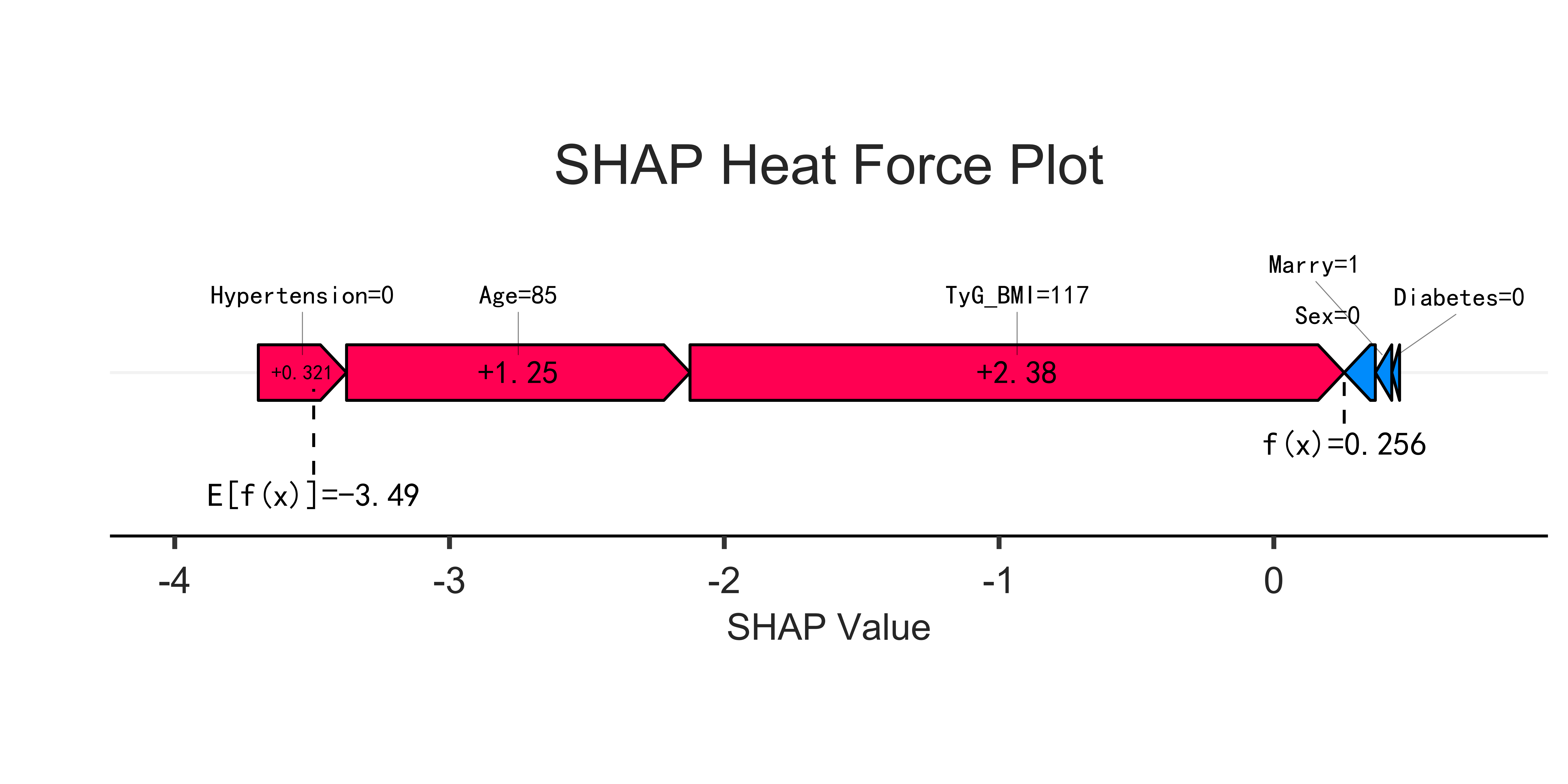


**C Train F Internal Test**


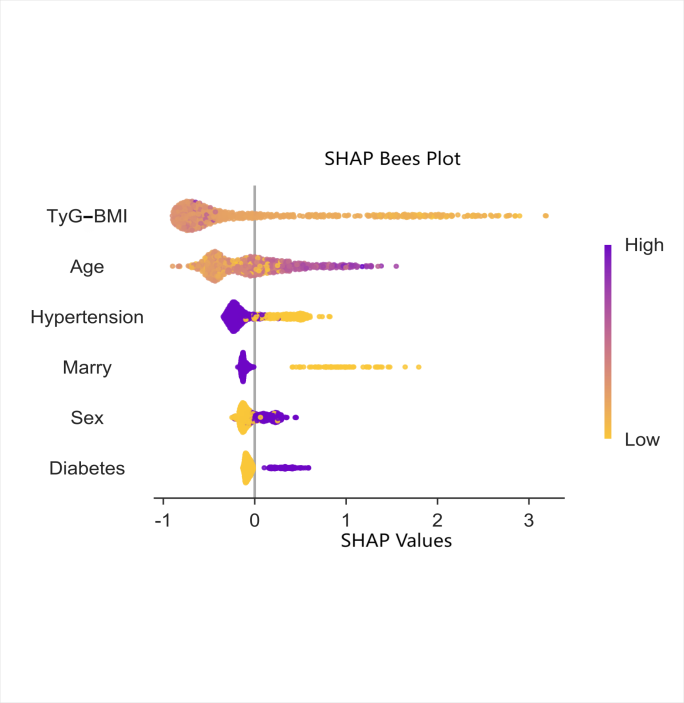

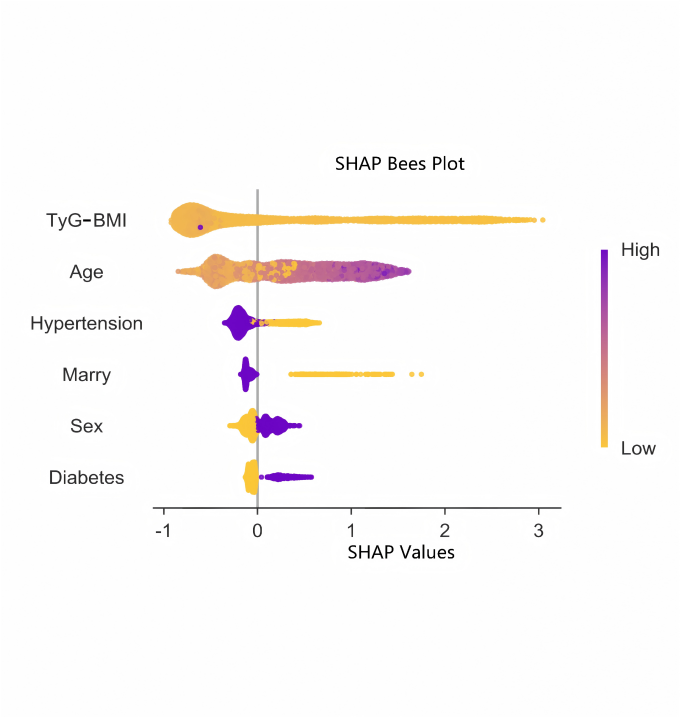


Legend:

Panels A and D show SHAP importance plots; panels B and E show SHAP force plots; panels C and F show SHAP beeswarm plots for the training and internal validation sets, respectively. SHAP values quantify the contribution of each feature to the model prediction.

Abbreviation: SHAP, Shapley Additive Explanations.

**Supplementary Figure S4： Associations of selected clinical risk factors with the CVD-SRS in the training and internal validation sets**

**Train Internal Test**


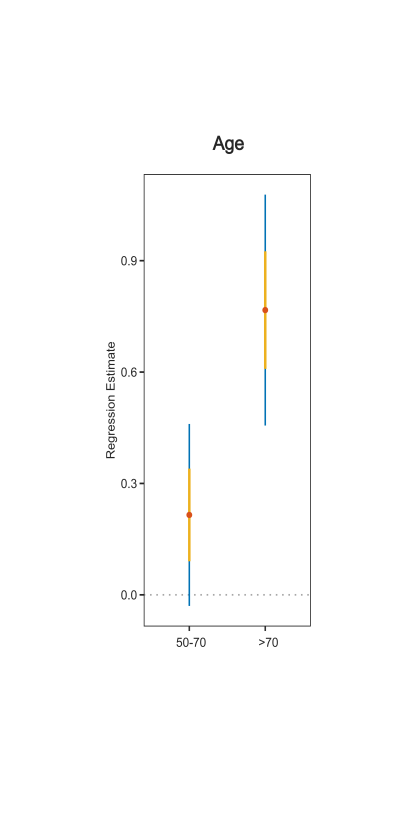

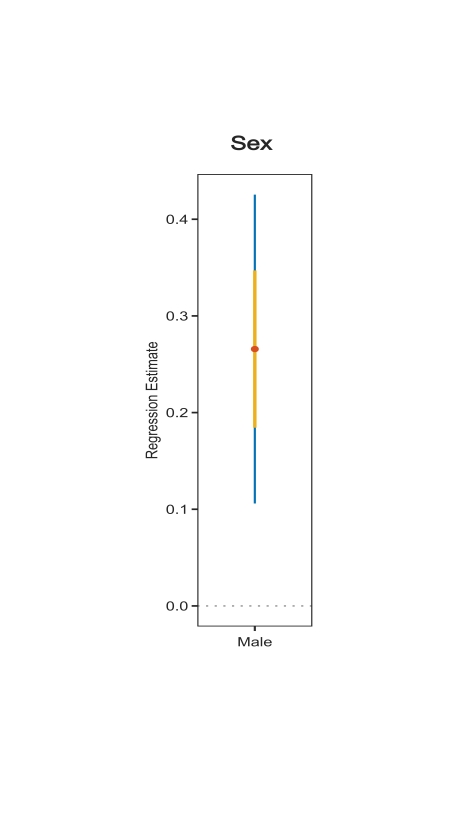

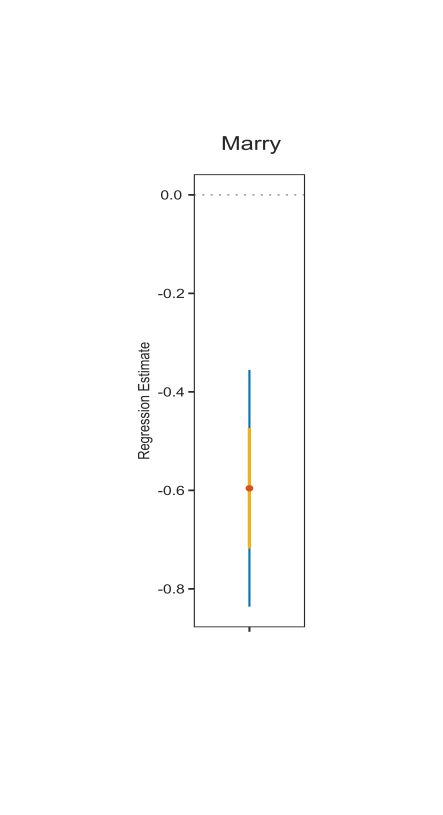

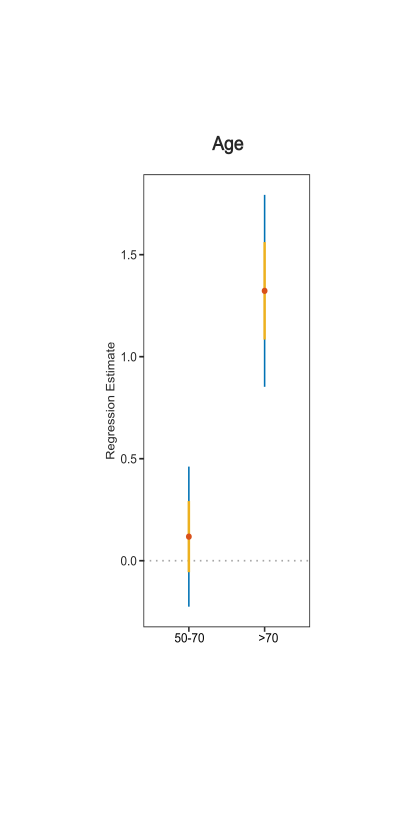

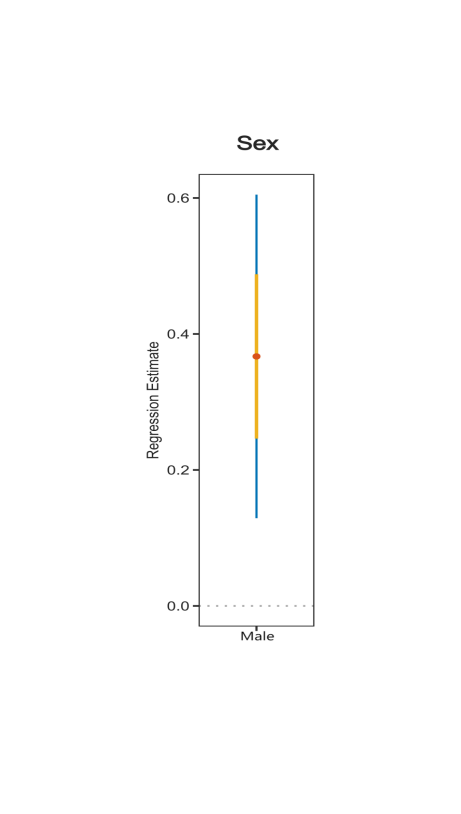

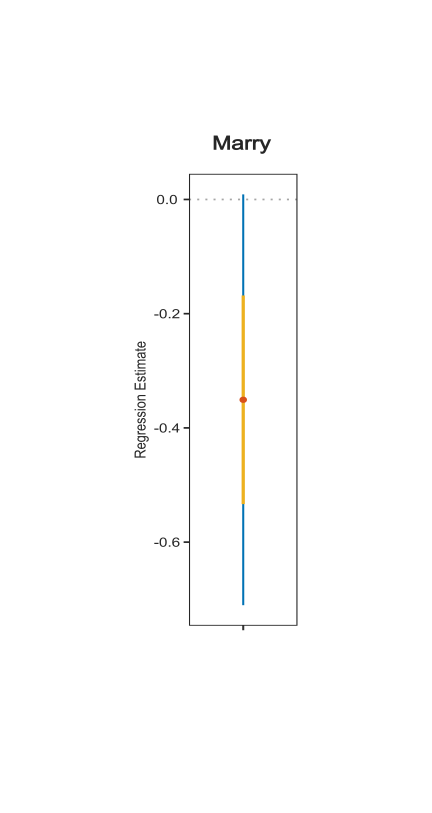


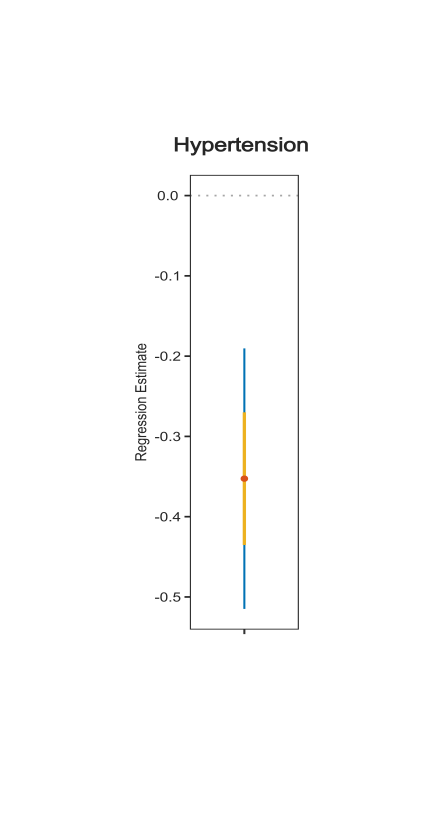

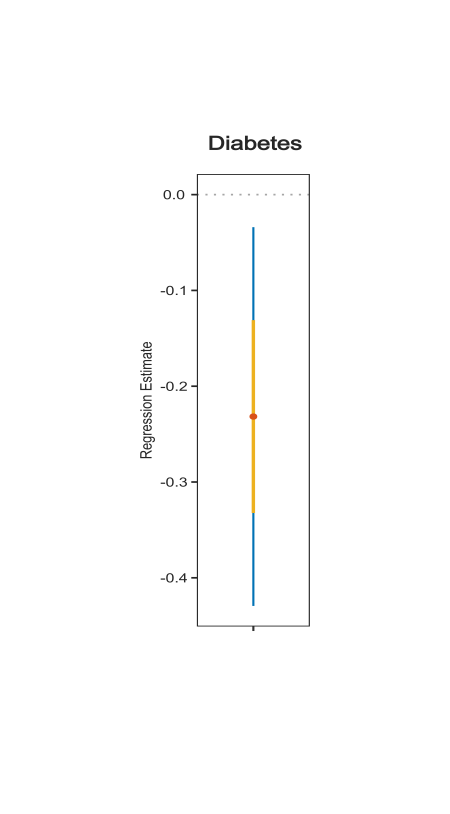

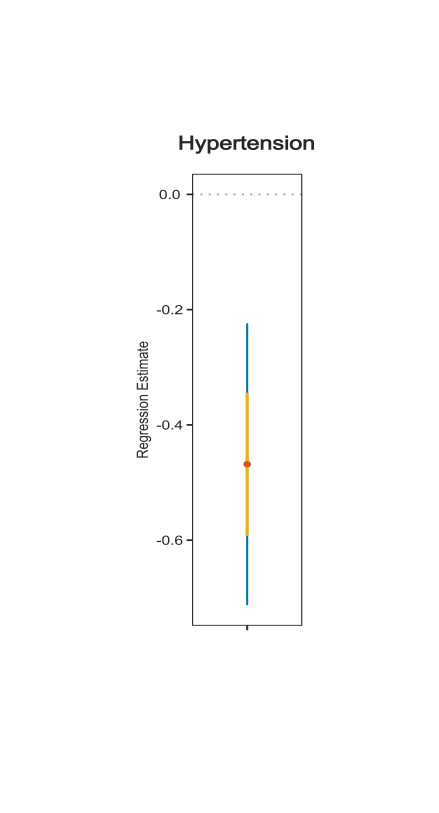

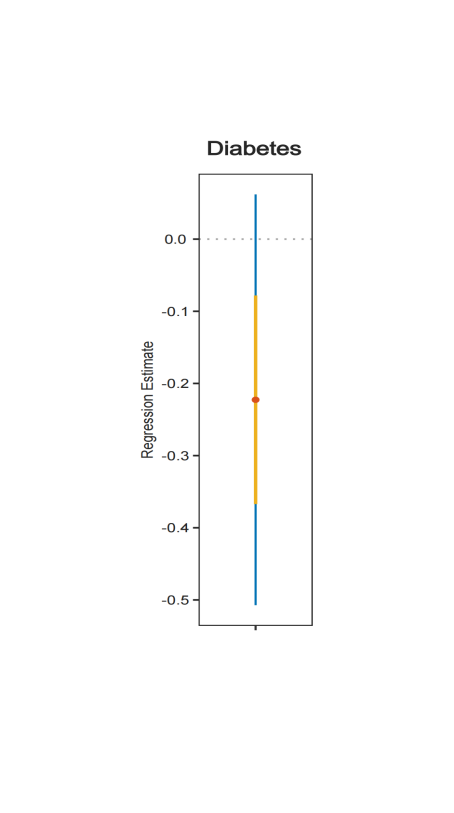


Legend:

Regression estimates and 95% confidence intervals are shown for selected risk factors associated with the CVD-SRS in the training and internal validation sets.

Abbreviation: CVD-SRS, Cardiovascular Disease–Sarcopenia Risk Score.

**Supplementary Figure S5：Restricted cubic spline analysis of the association between the CVD-SRS and sarcopenia risk across cohorts**


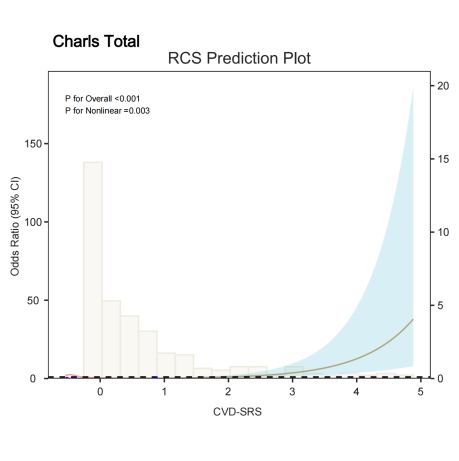

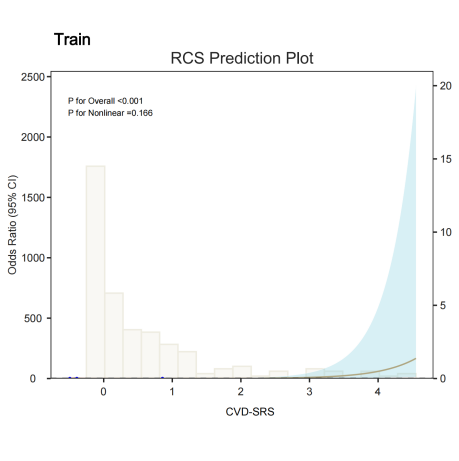
**
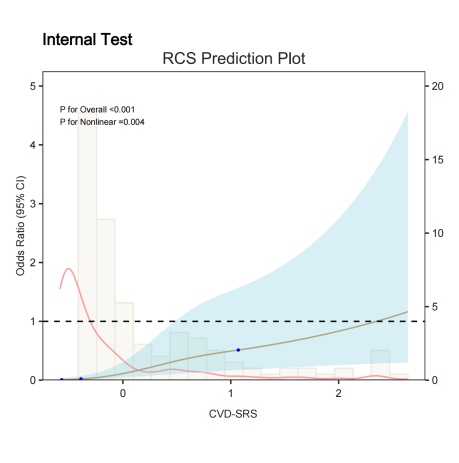
**
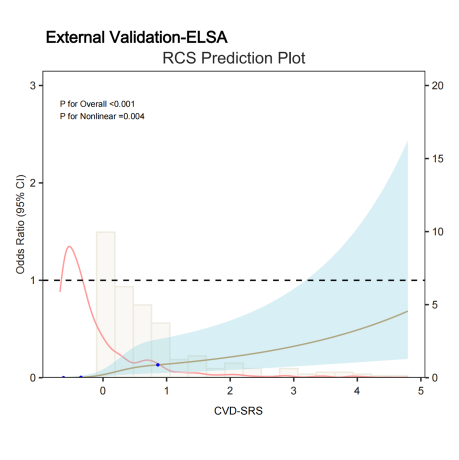
**
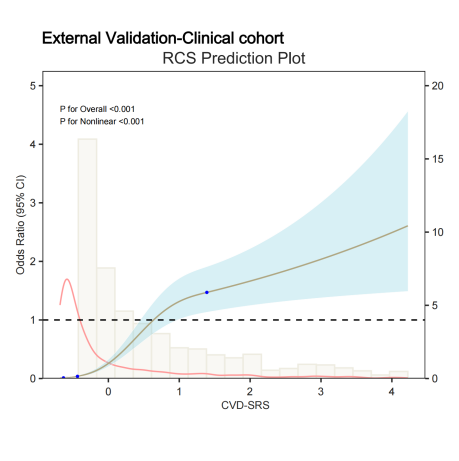
**

Legend:

Restricted cubic spline plots show the association between the continuous CVD-SRS and sarcopenia risk in the CHARLS total sample, training set, internal validation set, ELSA external validation cohort, and clinical external validation cohort. Shaded areas indicate 95% confidence intervals.

Abbreviation: CVD-SRS, Cardiovascular Disease–Sarcopenia Risk Score.
